# Shield synthesis

**DOI:** 10.1007/s10703-017-0276-9

**Published:** 2017-09-25

**Authors:** Bettina Könighofer, Mohammed Alshiekh, Roderick Bloem, Laura Humphrey, Robert Könighofer, Ufuk Topcu, Chao Wang

**Affiliations:** 1 IAIK, Graz University of Technology, Graz, Austria; 2 University of Texas at Austin, Austin, TX USA; 3 Control Science Center of Excellence, AFRL, Wright-Patterson AFB, Fairborn, OH USA; 4 Department of ECE, Virginia Tech, Blacksburg, VA 24061 USA

**Keywords:** Synthesis, Runtime reinforcement, Games, Human factors, UAV

## Abstract

*Shield synthesis* is an approach to enforce safety properties at runtime. A shield monitors the system and corrects any erroneous output values instantaneously. The shield deviates from the given outputs as little as it can and recovers to hand back control to the system as soon as possible. In the first part of this paper, we consider shield synthesis for reactive hardware systems. First, we define a general framework for solving the shield synthesis problem. Second, we discuss two concrete shield synthesis methods that automatically construct shields from a set of *safety* properties: (1) *k-stabilizing* shields, which guarantee recovery in a finite time. (2) *Admissible* shields, which attempt to work with the system to recover as soon as possible. Next, we discuss an extension of *k*-stabilizing and admissible shields, where erroneous output values of the reactive system are corrected while liveness properties of the system are preserved. Finally, we give experimental results for both synthesis methods. In the second part of the paper, we consider shielding a human operator instead of shielding a reactive system: the outputs to be corrected are not initiated by a system but by a human operator who works with an autonomous system. The challenge here lies in giving simple and intuitive explanations to the human for any interferences of the shield. We present results involving mission planning for unmanned aerial vehicles.

## Introduction

Technological advances enable the development of increasingly sophisticated systems. Smaller and faster microprocessors, wireless networking, and new theoretical results in areas such as machine learning and intelligent control are paving the way for transformative technologies across a variety of domains—self-driving cars that have the potential to reduce accidents, traffic, and pollution; and unmanned systems that can safely and efficiently operate on land, under water, in the air, and in space. However, in each of these domains, concerns about safety are being raised [13, 27]. Specifically, there is a concern that due to the complexity of such systems, traditional test and evaluation approaches will not be sufficient for finding errors, and alternative approaches such as those provided by formal methods are needed [28].

Formal methods are often used to verify systems at design time, but this is not always realistic. Some systems are simply too large to be fully verified. Others, especially systems that operate in rich dynamic environments or those that continuously adapt their behavior through methods such as machine learning, cannot be fully modeled at design time. Still others may incorporate components that have not been previously verified and cannot be modeled, e.g., pre-compiled code libraries. Also, even systems that have been fully verified at design time may be subject to external faults such as those introduced by unexpected hardware failures or human inputs. One way to address this issue is to model nondeterministic behaviours (such as faults) as disturbances, and to verify the system with respect to this disturbance model [29]. However, it may be impossible to model all potential unexpected behavior at design time.

An alternative in such cases is to perform *runtime verification* to detect violations of specified properties while a system is executing [24]. An extension of this idea is to perform *runtime enforcement* of specified properties, in which violations are not only detected but also overwritten in such a way that specified properties are maintained.

**Fig. 1:**
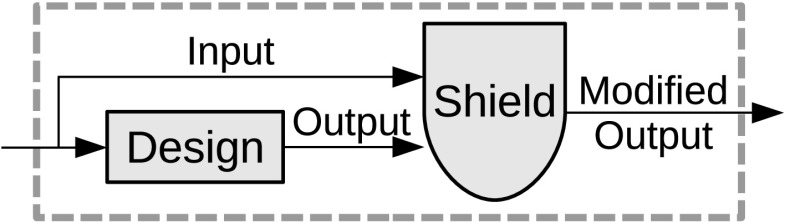
Attaching a safety shield

In this paper, we discuss a general approach for runtime enforcement called *shield synthesis*. From the specified properties, we automatically construct a component, called the shield, that monitors the input/output of the system and instantaneously overwrites incorrect outputs as illustrated in Fig. 1.

A shield must ensure both *correctness*, i.e., it corrects system outputs such that all properties are always satisfied, as well as *minimum deviation*, i.e., it deviates from system outputs only if necessary and as rarely as possible. The latter requirement is important because the system may satisfy additional noncritical properties that are not considered by the shield but should be retained as much as possible. Shield synthesis is a promising new direction for synthesis in general, because it uses the strengths of reactive synthesis while avoiding its weaknesses—the set of critical properties can be small and relatively easy to specify regardless of the implementation details of a complex system—which implies scalability and usability.

In the first part of this paper, we consider shield synthesis for reactive hardware systems. Here, we first define a general framework for solving the shield synthesis problem. Second, we discuss two concrete shield synthesis methods to automatically construct a shield from a set of safety properties. The resulting shields are called *k*-stabilizing shields and admissible shields.

*k* *-stabilizing* shields guarantee recovery in a finite time. Since we are given a safety specification, we can identify wrong outputs, that is, outputs after which the specification is violated (more precisely, after which the environment can force the specification to be violated). A wrong trace is then a trace that ends in a wrong output. *k*-stabilizing shields modify the outputs so that the specification always holds, but that such deviations last for at most *k* consecutive steps after a wrong output.

*Admissible* shields overcome the following shortcoming of *k*-stabilizing shields: The *k*-stabilizing shield synthesis problem is unrealizable for many safety-critical systems, because a finite number of deviations cannot be guaranteed. To address this issue, admissible shields guarantee the following: (1) For any wrong trace, if there is a finite number *k* of steps within which the recovery phase can be guaranteed to end, an admissible shield takes an *adversarial* view on the system and will always achieve this. Admissible shields are subgame optimal and guarantee to end the recovery phase for any state for the smallest *k* possible if such a *k* exists for that state. (2) The shield is *admissible* in the following sense: for any state in which there is no such number *k*, it takes a *collaborative* view on the system and always picks a deviation that is optimal in that it ends the recovery phase as soon as possible for some possible future inputs. As a result, admissible shields work well in settings in which finite recovery cannot be guaranteed, because they guarantee correctness and may well end the recovery period if the system does not pick adversarial outputs.

*k*-stabilizing shields and admissible shields enforce critical safety properties and ensure minimum deviation, such that other noncritical properties of the system that are not considered by the shield are retained as much as possible. In addition to critical safety properties, many systems must also meet critical liveness properties. However, a challenge for enforcing liveness properties using shields is that liveness property violations cannot be detected at any finite point in time (at any point, the property may still be satisfied in the future). Due to the minimum deviation property of shields, a shield would have to delay enforcing a liveness property as long as possible, and since liveness properties can always be satisfied at some point in the future, the shield in practice would never enforce the liveness property. So rather than enforcing liveness properties, we focus on retaining liveness properties under the assumption that the shielded system satisfies them. We therefore conclude the first part of this paper with an extension of the *k*-stabilizing and the admissible shield synthesis procedure that allows liveness-preserving corrections of the system’s output.

In the second part of the paper, we consider shielding a human operator who works with an autonomous system instead of shielding a reactive system: the outputs to be corrected are not initiated by a system but by a human operator. When shielding human operators we attach the shield *before* the operator. We call this type of shield a *preemptive shield*. The shield acts each time the operator is to make a decision and provides a list of safe outputs. This list restricts the choices for the operator. Additionally, when shielding a human operator, it is necessary to provide simple and intuitive explanations to the operator for any interferences of the shield. We call shields able to provide such explanations *explanatory shields*. We motivate the need for shielding a human operator via a case study involving mission planning for an unmanned aerial vehicle (UAV).

### Outline

The remainder of this paper is organized as follows. First, we establish notation in Sect. 2. In Sect. 3 we discuss shield synthesis for reactive hardware systems. We begin this section by using an example to illustrate the technical challenges and our solution approach in Sect. 3.1. We formalize the problem in a general framework for shield synthesis in Sect. 3.2. In Sects. 3.3, 3.4, 3.5, and 3.6 we define and describe the synthesis procedure for *k*-stabilizing shields and for admissible shields. Section 3.7 describes an alternative construction for *k*-stabilizing and admissible shields, and Sect. 3.8 discusses liveness-preserving shielding. To conclude the first part of the paper, we provide experimental results for both shield synthesis approaches in Sect. 3.9. In Sect. 4 we consider shielding a human operator instead of shielding a reactive system. In this setting, we discuss preemptive shields in Sect. 4.1 and explanatory shields in Sect. 4.2. We conclude the second part of the paper with a case study on UAV mission planning in Sect. 4.3 Finally, we give an overview on related work in Sect. 5 and conclude in Sect. 6.

## Preliminaries

We denote the Boolean domain by $\mathbb {B}=\{\top ,\bot \}$, the set of natural numbers by $\mathbb {N}$, and abbreviate $\mathbb {N}\cup \{\infty \}$ by $\mathbb {N}^\infty$. The set of finite (infinite) words over an alphabet $\varSigma$ is denoted by $\varSigma ^*$ ($\varSigma ^\omega$), and $\varSigma ^{\infty } = \varSigma ^* \cup \varSigma ^\omega$. We will also refer to words as *(execution) traces*. We write $|\overline{\sigma }|$ for the length of a trace $\overline{\sigma }\in \varSigma ^*$. A set $L\subseteq \varSigma ^\infty$ of words is called a *language*. We denote the set of all languages as $\mathcal {L}= 2^{\varSigma ^\infty }$. We consider a finite set $I=\{i_1,\ldots ,i_m\}$ of Boolean inputs and a finite set $O=\{o_1,\ldots ,o_n\}$ of Boolean outputs. The input alphabet is $\varSigma _I=2^I$, the output alphabet is $\varSigma _O=2^O$, and $\varSigma =\varSigma _I\times \varSigma _O$. For ${\overline{\sigma _I}}= x_0 x_1 \ldots \in \varSigma _I^\infty$ and ${\overline{\sigma _O}}= y_0 y_1 \ldots \in \varSigma _O^\infty$, we write ${\overline{\sigma _I}}|| {\overline{\sigma _O}}$ for the composition $(x_0,y_0) (x_1,y_1) \ldots \in \varSigma ^\infty$.

### Reactive systems

A *Mealy machine* (a reactive system, also called a design) is a 6-tuple $\mathcal {D}= (Q, q_0, \varSigma _I, \varSigma _O, \delta , \lambda )$, where $Q$ is a finite set of states, $q_0\in Q$ is the initial state, $\delta : Q\times \varSigma _I\rightarrow Q$ is a complete transition function, and $\lambda : Q\times \varSigma _I\rightarrow \varSigma _O$ is a complete output function. Given the input trace ${\overline{\sigma _I}}= x_0 x_1 \ldots \in \varSigma _I^\infty$, the system $\mathcal {D}$ produces the output trace ${\overline{\sigma _O}}= \mathcal {D}({\overline{\sigma _I}}) = \lambda (q_0, x_0) \lambda (q_1, x_1) \ldots \in \varSigma _O^\infty$, where $q_{i+1} = \delta (q_i, x_i)$ for all $i \ge 0$. The set of words produced by $\mathcal {D}$ is denoted $L(\mathcal {D}) = \{{\overline{\sigma _I}}|| {\overline{\sigma _O}}\in \varSigma ^\infty \mid \mathcal {D}({\overline{\sigma _I}}) = {\overline{\sigma _O}}\}$.

Let $\mathcal {D}= (Q, q_0, \varSigma _I, \varSigma _O, \delta , \lambda )$ and $\mathcal {D}' = (Q', q_0', \varSigma , \varSigma _O, \delta ', \lambda ')$ be two reactive systems. A serial composition of $\mathcal {D}$ and $\mathcal {D}'$ is realized if the input and output of $\mathcal {D}$ are fed to $\mathcal {D}'$. We denote such composition as $\mathcal {D}\circ \mathcal {D}'=(\hat{Q}, \hat{q_0}, \varSigma _I, \varSigma _O, \hat{\delta }, \hat{\lambda })$, where $\hat{Q} = Q\times Q'$, $\hat{q_0} = (q_0, q_0')$, $\hat{\delta }((q,q'),{\sigma _I}) = (\delta (q,{\sigma _I}), \delta '(q',({\sigma _I},\lambda (q,{\sigma _I}))))$, and $\hat{\lambda }((q,q'),{\sigma _I}) = \lambda '(q',({\sigma _I},\lambda (q,{\sigma _I})))$.

### Automata

An automaton *A* is a tuple $A = (Q, q_0, \varSigma , \delta , Acc)$ , where $Q$ is a finite set of states, $q_0\subseteq Q$ is the initial state, $\delta : Q\times \varSigma \rightarrow Q$ is the transition function, and *Acc* is the acceptance condition. The *run* induced by trace $\overline{\sigma }= \sigma _0 \sigma _1 \ldots \in \varSigma ^\omega$ is the state sequence $\overline{q} = q_0 q_1 \ldots$ such that $q_{i+1} = \delta (q_i, \sigma _i)$. *A* accepts a trace $\overline{\sigma }$ if its run $\overline{q}$ is accepting $(Acc(\overline{q}) = \top )$; its language *L*(*A*) consists of the set of traces it accepts.

### Acceptance conditions

The specifications we use are automata and we synthesize a system that realizes a given specification using games. Both automata and games can have the following acceptance conditions. Let *Q* be a set of states, an acceptance condition is a predicate $Acc : Q^\omega \rightarrow \mathbb {B}$, mapping infinite runs $\overline{q}$ to $\top$ or $\bot$ (accepting and not accepting, or winning and losing, respectively).

A *safety* acceptance condition is defined by a function $Acc(\overline{q}) = \top$ iff $\forall i\ge 0 {{\mathrm{\mathbin {.}}}}q_i \in F$, where $\overline{q} = q_0 q_1 \ldots$ and $F \subseteq Q$ is the set of safe states. The *reachability* acceptance condition is $Acc(\overline{q}) = \top$ iff $\exists i\ge 0 {{\mathrm{\mathbin {.}}}}q_i \in F$, where $F \subseteq Q$ is the set of reachable states. The *Büchi* acceptance condition is $Acc(\overline{q}) = \top$ iff $\inf (\overline{q}) \cap F \ne \emptyset$, where $F \subseteq Q$ is the set of accepting states and $\inf (\overline{q})$ is the set of states that occur infinitely often in $\overline{q}$. We abbreviate the Büchi condition as $\mathcal {B}(F)$. A *Generalized Reactivity 1* (GR(1)) acceptance condition is a predicate $\bigwedge _{i=1}^{m} \mathcal {B}(E_{i}) \rightarrow \bigwedge _{i=1}^{n} \mathcal {B}(F_{i})$, with $E_i \subseteq Q$ and $F_i \subseteq Q$. The acceptance condition is a *generalized Büchi* acceptance condition if $m = 0$. A *Streett* acceptance condition with *k* pairs is a predicate $\bigwedge _{i=1}^{k}\big ( \mathcal {B}(E_{i}) \rightarrow \mathcal {B}(F_{i} ) \big )$.

### Specifications

A *specification* $\varphi$ is a set $L(\varphi ) \subseteq \varSigma ^\infty$ of allowed traces. A design $\mathcal {D}$ *realizes* $\varphi$, denoted by $\mathcal {D}\models \varphi$, iff $L(\mathcal {D}) \subseteq L(\varphi )$. $\varphi$ is *realizable* if there exists a design $\mathcal {D}$ that realizes it.

A property $\varphi ^s$ defines a *safety* property [2] if finite traces that do not satisfy $\varphi ^s$ cannot be extended to traces that satisfy $\varphi ^s$, i.e., $\forall \overline{\sigma }\in \varSigma ^* {{\mathrm{\mathbin {.}}}}(\overline{\sigma }\not \models \varphi ^s \rightarrow (\forall \overline{\sigma }' \in \varSigma ^\infty {{\mathrm{\mathbin {.}}}}(\overline{\sigma }\cdot \overline{\sigma }') \not \models \varphi ^s))$. The intuition is that a safety property states that “something bad” must never happen. If $\varphi ^s$ does not hold for a trace, then at some point some “bad thing” must have happened and such a “bad thing” must be irremediable. We represent a pure safety specification $\varphi ^s$ by a safety automaton $\varphi ^s = (Q, q_0, \varSigma , \delta , F)$, where $F\subseteq Q$ is a set of safe states. A trace $\overline{\sigma }$ (of a design $\mathcal {D}$) *satisfies* $\varphi ^s$ if the induced run is accepting. The *language* $L(\varphi ^s)$ is the set of all traces satisfying $\varphi$.

A property $\varphi ^l$ defines a *liveness* property [2] if every finite trace can be extended to an infinite trace that satisfies $\varphi ^l$, i.e., $\forall \overline{\sigma }\in \varSigma ^* {{\mathrm{\mathbin {.}}}}\exists \overline{\sigma }' \in \varSigma ^\omega {{\mathrm{\mathbin {.}}}}(\overline{\sigma }\cdot \overline{\sigma }') \models \varphi ^l$. Informally, a liveness property stipulates that a “good thing” happens during execution eventually.

### Games

A (2-player, alternating) *game* is a tuple $\mathcal {G}= (G, g_0, \varSigma _I, \varSigma _O, \delta , Acc)$, where $G$ is a finite set of game states, $g_0\in G$ is the initial state, $\delta : G\times \varSigma _I\times \varSigma _O\rightarrow G$ is a complete transition function, and $Acc: G^\omega \rightarrow \mathbb {B}$ is a winning condition. The game is played by two players: the system and the environment. In every state $g\in G$ (starting with $g_0$), the environment first chooses an input letter ${\sigma _I}\in \varSigma _I$, and then the system chooses some output letter ${\sigma _O}\in \varSigma _O$. This defines the next state $g' = \delta (g,{\sigma _I}, {\sigma _O})$, and so on. Thus, a finite or an infinite word over $\varSigma$ results in a finite or an infinite *play*, a sequence $\overline{g} = g_0 g_1 \ldots$ of game states. A play is *won* by the system iff $Acc(\overline{g})$ is $\top$.

A deterministic (memoryless) *strategy* for the environment is a function $\rho _e: G\rightarrow \varSigma _I$. A non-det. (memoryless) *strategy* for the system is a relation $\rho _s: G\times \varSigma _I\rightarrow 2^{\varSigma _O}$ and a det. (memoryless) *strategy* for the system is a function $\rho _s: G\times \varSigma _I\rightarrow \varSigma _O$. A strategy $\rho _s$ is *winning* for the system if, *for all* strategies $\rho _e$ of the environment, the play $\overline{g}$ that is constructed when defining the outputs using $\rho _e$ and $\rho _s$ satisfies $Acc(\overline{g})$. The *winning region* *W* is the set of states from which a winning strategy for the system exists. A *counterstrategy* is a winning strategy for the environment from $g_0$. A counterstrategy exists if $g_0 \not \in W$. Let $\mathcal {G}= (G, g_0, \varSigma _I, \varSigma _O, \delta , F)$ be a safety game with winning region *W*. If $g_0\not \in W$, a counterstrategy can be computed by solving a reachability game $\mathcal {G}' = (G, g_0, \varSigma _I, \varSigma _O, \delta , Q \backslash F)$. In safety, reachability and Büchi games, both players have memoryless winning strategies, whereas for GR(1), Streett and generalized Büchi games, finite-memory strategies are necessary for the system.

It is easy to transform a safety specification $\varphi ^s$ into a safety game such that a trace satisfies the specification iff the corresponding play is won. A finite trace $\overline{\sigma }\in \varSigma ^*$ is *wrong* if the corresponding play $\overline{g}$ contains a state outside the winning region *W*. Otherwise $\overline{\sigma }$ is called *correct*. An *output* is called *wrong* if it makes a trace wrong; i.e., given $\varphi ^s$, a trace $\overline{\sigma }\in \varSigma ^*$, ${\sigma _I}\in \varSigma _I$, and ${\sigma _O}\in \varSigma _O$, ${\sigma _O}$ is wrong iff $\overline{\sigma }$ is correct, but $\overline{\sigma }\cdot ({\sigma _I},{\sigma _O})$ is wrong. Otherwise ${\sigma _O}$ is called *correct*.

### Comparing strategies

First, we compare non-deterministic winning strategies of the system by comparing the *behaviours* that they allow [3]. If $\rho$ is a strategy and *g* is a state of $\mathcal {G}$ from which $\rho$ is winning then $Beh(\mathcal {G}, g, \rho )$ is the set of all plays starting in *g* and respecting $\rho$. If $\rho$ is not winning from *g* then we put $Beh(\mathcal {G}, g, \rho )=\emptyset$. A strategy subsumes another strategy if it allows more behaviours, i.e., a strategy $\rho '$ is *subsumed* by $\rho$, which is denoted $\rho ' \sqsubseteq \rho$ if $Beh(\mathcal {G}, g, \rho ') \subseteq Beh(\mathcal {G}, g, \rho )$ for all $g \in G$. A strategy $\rho$ is *permissive* if $\rho ' \sqsubseteq \rho$ for every memoryless strategy $\rho '$.

Second, we compare deterministic strategies of the system in game states from which the system cannot force a win [19]. A system strategy $\rho _s$ is *cooperatively winning* if there exists an environment strategy $\rho _e$ such that the play $\overline{g}$ constructed by $\rho _e$ and $\rho _s$ satisfies $Acc(\overline{g})$. For a Büchi game $\mathcal {G}$ with accepting states *F*, consider a strategy $\rho _e$ of the environment, a strategy $\rho _s$ of the system, and a state $g\in G$. We define the distance $dist(g, \rho _e, \rho _s)=d$ if the play $\overline{g}$ defined by $\rho _e$ and $\rho _s$ reaches from *g* an accepting state that occurs infinitely often in $\overline{g}$ in *d* steps. If no such state is visited, we set $dist(g, \rho _e, \rho _s)=\infty$. Given two strategies $\rho _s$ and $\rho _s'$ of the system, we say that $\rho _s'$ *dominates* $\rho _s$ if: (i) for all $\rho _e$ and all $g\in G$, $dist(g,\rho _e,\rho _s')\le dist(g,\rho _e,\rho _s)$, and (ii) there exists $\rho _e$ and $g\in G$ such that $dist(g,\rho _e,\rho _s')< dist(g,\rho _e,\rho _s)$. A strategy is *admissible* if there is no strategy that dominates it.

## Shield synthesis for reactive systems

The goal of shield synthesis is to enforce a small set of safety properties at runtime, even if these properties may be violated by the reactive system, called the *design*. We synthesize a shield directly from the set of safety properties, and attach it to the design as illustrated in Fig. 1. The shield monitors the input/output of the design and corrects the erroneous output values instantaneously, but only if necessary and as infrequently as possible. In the next section, we consider an example of a simple traffic light controller to illustrate the challenges addressed by shield synthesis.

### Motivating example

Let us consider the example of a traffic light controller of two roads. There are red (r) or green (g) lights for both roads, i.e., $\varSigma = \varSigma _O= \{ \texttt {rr} , \texttt {rg} , \texttt {gr} , \texttt {gg} \}$. Although the traffic light controller interface is simple, the actual implementation can be complex. The controller may have to be synchronized with other traffic lights, and it can have input sensors for cars, buttons for pedestrians, and sophisticated algorithms to optimize traffic throughput based on all sensors, the time of the day, and even the weather. As a result, the actual design may become too complex to be formally verified.

**Fig. 2:**
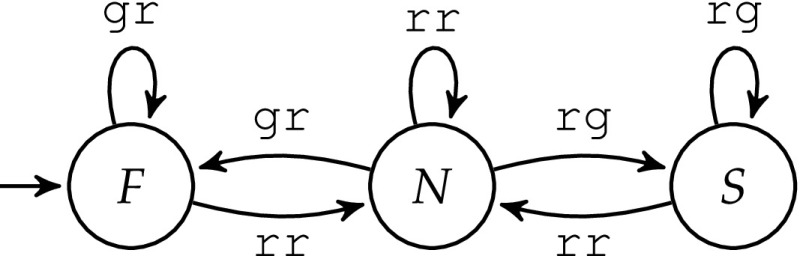
Traffic light specification

Suppose now that two safety properties are crucial and must be satisfied with certainty: (1) The output gg—meaning that both roads have green lights—is never allowed. (2) The output cannot change from gr to rg, or vice versa, without passing rr. These two properties serve as specification $\varphi$ for the shield, and can be expressed by the automaton shown in Fig. 2. Edges are labeled with the controller’s outputs for the two roads. *F* denotes the state where the first road has the green light, *S* denotes the state where the second road has the green light, and *N* denotes the state where both have red lights. There is also an error state, which is not shown. Missing edges lead to this error state, denoting forbidden situations, e.g., gg is never allowed. Although the automaton may not be a complete specification for a full traffic light system controller design, the corresponding shield can prevent catastrophic failures.

**Table 1:** Controller shielded by $\mathcal {S}_{A}$

Time step	1	2	3	4	5
Controller	rr	**gg**	**gr**	gr	rr
Shield $\mathcal {S}_{A}$	rr	*rg*	*rr*	gr	rr

**Table 2:** Controller shielded by $\mathcal {S}_{B}$

Time step	1	2	3	4	5
Controller	rr	**gg**	gr	gr	rr
Shield $\mathcal {S}_{B}$	rr	*rr*	gr	gr	rr

Tables 1 and 2 show how two different shields ($\mathcal {S}_A$ and $\mathcal {S}_B$, respectively) correct a sample output of a traffic light controller. Let us first consider Table 1. In time step 1, the controller sends the output rr which is accepted and passed on by the shield $\mathcal {S}_A$. In step 2, the controller sends gg, which violates $\varphi$. $\mathcal {S}_A$ has three options for a correction: changing the output from gg to either rg, gr, or rr. The shield $\mathcal {S}_A$ corrects the output to rg. In step 3, the controller gives the output gr. Since the traffic light cannot toggle from rg to gr according to $\varphi$, $\mathcal {S}_A$ changes the output to rr. Afterwards, the controller again sends gr and $\mathcal {S}_A$ is able to end the deviation and to pass on outputs from the controller until the next specification violation.

Let us analyse the behavior of the shield $\mathcal {S}_A$. First, the shield’s output was correct with respect to $\varphi$. Second, to ensure minimum deviation, $\mathcal {S}_A$ only deviated from the controller when a property violation became unavoidable. Finally, the shield ended deviation after 2 steps, and then handed back control to the traffic light controller.

In Table 2 we use the shield $\mathcal {S}_B$, which corrects the controller’s output in step 2 to rr. This time if the controller sends gr in step 3, the shield can give the same output as the controller immediately. If we compare the shields, $\mathcal {S}_B$ ends the deviation phase faster than $\mathcal {S}_A$. Hence, we prefer the behavior induced by $\mathcal {S}_B$.

**Fig. 3:**
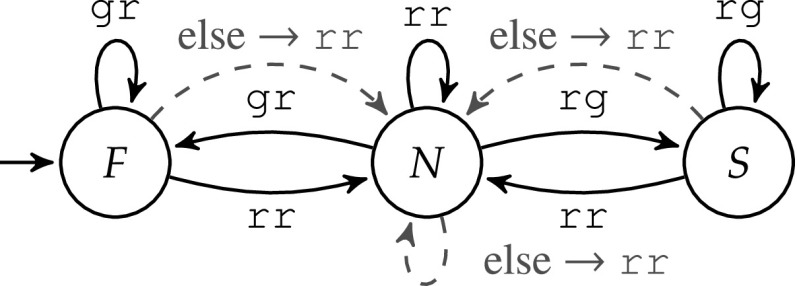
Traffic light shield $\mathcal {S}_B$

The behavior of the shield $\mathcal {S}_B$ is illustrated in Fig. 3. Edges are labeled with the inputs of the shield. Red dashed edges denote situations where the output of the shield is different from its inputs. The modified output is written after the arrow. For all non-dashed edges, the input is just copied to the output.

The challenge in shield synthesis lies in the fact that we do not know the future inputs/outputs of the design. The question is, without knowing what the future inputs/outputs are, how should the shield correct bad behavior of the design to avoid unnecessarily large deviation in the future? For instance, the correction of shield $\mathcal {S}_A$ in step 2 was suboptimal, since it caused a deviation for 2 steps instead of 1. In the next section, we discuss a general framework of shield synthesis for reactive systems.

### Definition of shields

A shield reads the input and output of a design as shown in Fig. 1. In this section, we formally define the two desired properties: *correctness* and *minimum deviation*.

#### The correctness property

By correctness, we refer to the property that the shield corrects any design’s output such that a given safety specification is satisfied. Formally, let $\varphi$ be a safety specification and $\mathcal {S}= (Q', q_0', \varSigma , \varSigma _O, \delta ', \lambda ')$ be a Mealy machine. We say that $\mathcal {S}$ *ensures correctness* if for any design $\mathcal {D}= (Q, q_0, \varSigma _I, \varSigma _O, \delta , \lambda )$, it holds that $(\mathcal {D}\circ \mathcal {S}) \models \varphi$.

Since a shield must work for any design, the synthesis procedure does not consider the design’s implementation. This property is crucial because the design may be unknown or too complex to analyze. On the other hand, the design may satisfy additional (noncritical) specifications that are not specified in $\varphi$ but should be retained as much as possible (i.e., as long as these additional properties are not in conflict with the critical ones).

#### The minimum deviation property

Minimum deviation requires a shield to deviate only if necessary, and as infrequently as possible. To ensure minimum deviation, a shield can only deviate from the design if a property violation becomes unavoidable. Given a safety specification $\varphi$, a Mealy machine $\mathcal {S}$ *does not deviate unnecessarily* if for any design $\mathcal {D}$ and any trace ${\overline{\sigma _I}}||{\overline{\sigma _O}}$ of $\mathcal {D}$ that is not wrong, we have that $\mathcal {S}({\overline{\sigma _I}}||{\overline{\sigma _O}}) = {\overline{\sigma _O}}$. In other words if $\mathcal {D}$ does not violate $\varphi$, $\mathcal {S}$ keeps the output of $\mathcal {D}$ intact.

##### Definition 1

Given a specification $\varphi$, a Mealy machine $\mathcal {S}$ is a *shield* if for any design $\mathcal {D}$, it holds that $(\mathcal {D}\circ \mathcal {S}) \models \varphi$ and $\mathcal {S}$ does not deviate from $\mathcal {D}$ unnecessarily.

Ideally, shields end phases of deviation as soon as possible, recovering quickly. This property leaves room for interpretation. Different types of shields differentiate on how this property is realized. In the next sections we will discuss *k*-stabilizing shields and admissible shields.

### *k*-stabilizing shields

**Fig. 4:**
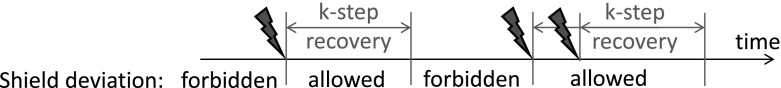
Recovery phases of *k*-stabilizing shields

We assume that through transmission errors an arbitrary number of correct outputs by the design $\mathcal {D}$ are replaced by wrong outputs; i.e., by outputs after which a property violation becomes unavoidable (in the worst case over future inputs). After each wrong output, a *k*-stabilizing shield $\mathcal {S}$ enters a recovery phase and is allowed to deviate from the design’s outputs for at most *k* consecutive time steps, including the current step. This is illustrated in Fig. 4. Wrong outputs are indicated by lightning.

We will now define *k*-stabilizing shields.

#### Definition 2

Let $\varphi$ be a safety specification and let $\overline{\sigma }= ({\overline{\sigma _I}}||{\overline{\sigma _O}}) \in \varSigma ^{\omega }$ be a correct trace. A shield $\mathcal {S}$ *adversely k-stabilizes* $\overline{\sigma }$ if, for any trace $\overline{\sigma }^f = ({\overline{\sigma _I}}||{\overline{\sigma _O}}^f) \in \varSigma ^{\omega }$ in which for any *i* with ${\overline{\sigma _O}}[i]\ne {\overline{\sigma _O}}^f[i]$ it holds that $({\overline{\sigma _I}}[0\dots i-1] || {\overline{\sigma _O}}[0\dots i-1]) \cdot ({\overline{\sigma _I}}[i], {\overline{\sigma _O}}[i]^f)$ is wrong and $E=\{i \mid {\overline{\sigma _O}}[i] \ne {\overline{\sigma _O}}^f[i]\}$ we have

$$\begin{aligned}&{\overline{\sigma _O}}^\star =\mathcal {S}({\overline{\sigma _I}}, {\overline{\sigma _O}}^f),\\&\quad ({\overline{\sigma _I}}||{\overline{\sigma _O}}^\star )\models \varphi \text { and}\\&\quad \forall j {{\mathrm{\mathbin {.}}}}{\overline{\sigma _O}}^\star [j] \ne {\overline{\sigma _O}}^f[j] \rightarrow \exists i\in E. j-i \le k. \end{aligned}$$

Substituting an arbitrary number of outputs in ${\overline{\sigma _O}}$ by wrong outputs results in a new trace $\overline{\sigma }^f$. *E* denotes the indices of outputs in $\overline{\sigma }^f$ that are wrong. After any wrong output ${\overline{\sigma _O}}^f[i]$ with $i \in E$, the output of the shield ${\overline{\sigma _O}}^\star$ and the output of the design ${\overline{\sigma _O}}^f$ are allowed to deviate for at most *k* consecutive time steps.

Note that it is not always possible to adversely *k*-stabilize any finite trace for a given *k* or even for any *k*.

#### Definition 3

(*k-Stabilizing Shields*[8]) A shield $\mathcal {S}$ is *k*-stabilizing if it adversely *k*-stabilizes any finite trace.

A *k*-stabilizing shield guarantees to deviate from outputs of the design for at most *k* steps after each wrong output and to produce a correct trace. To understand the intuition behind adversely *k*-stabilizing a trace, suppose we take the point of view that the design produces some wrong trace $\overline{\sigma }^f = ({\overline{\sigma _I}}||{\overline{\sigma _O}}^f)$ but intended to produce some correct trace $\overline{\sigma }= ({\overline{\sigma _I}}||{\overline{\sigma _O}})$. For each wrong output of ${\overline{\sigma _O}}^f$, the shield must “guess” what correct output the design intended in order to produce some correct trace $\overline{\sigma }^\star = ({\overline{\sigma _I}}||{\overline{\sigma _O}}^\star )$. If the shield “guesses” a particular output incorrectly, it may have to deviate from subsequent outputs of the design that would have been correct in ${\overline{\sigma _O}}$ in order to meet the specification. The term *adversely* *k*-stabilizing means that such periods of deviation will last for at most *k* steps for *any* intended trace ${\overline{\sigma _O}}$ of the design, i.e., even if ${\overline{\sigma _O}}^\star \ne {\overline{\sigma _O}}$.

### Synthesizing *k*-stabilizing shields

**Fig. 5:**
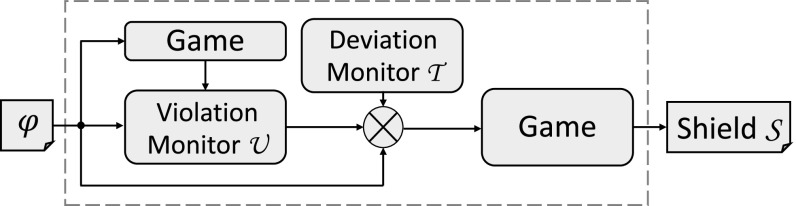
Outline of the shield synthesis procedure

The flow of our synthesis procedure for *k*-stabilizing shields is illustrated in Fig. 5. Let $$\varphi _1,\ldots ,\varphi _l$$ be the critical safety properties, where each $$\varphi _i$$ is represented as an automaton $$\varphi _i = (Q_i, q_{0,i}, \varSigma , \delta _i,$$ $$F_i)$$. The synchronous product $$\varphi = (Q, q_{0}, \varSigma , \delta ,$$ *F*) of these automata is again a safety automaton. Starting from $$\varphi $$, our shield synthesis procedure consists of five steps.

*Step 1 Constructing the violation monitor* $\mathcal {U}$: From $\varphi$ we build an automaton $\mathcal {U} = (U, u_0, \varSigma , \delta ^u)$ to monitor property violations by the design. The goal is to identify the latest point in time from which a specification violation can still be corrected with a deviation by the shield. This constitutes the start of the *recovery* phase, in which the shield is allowed to deviate from the design. The violation monitor $\mathcal {U}$ observes the design from all states the design could reach under the current input and a correct output. We note that when multiple states are being monitored, if the design’s output is wrong from all monitored states, $\mathcal {U}$ monitors all states the design could reach from all currently monitored states under the current input. If the design’s output is correct from one or more currently monitored states, it only continues monitoring states reachable from those monitored states under the design’s output.

The first phase of the construction (Step 1-a) of $\mathcal {U}$ considers $\varphi = (Q, q_{0}, \varSigma , \delta ,F)$ as a *safety game* and computes its winning region $W\subseteq F$ so that every reactive system $\mathcal {D}\models \varphi$ must produce outputs such that the next state of $\varphi$ stays in *W*. Only in cases in which the next state of $\varphi$ is outside of *W* is the shield allowed to interfere.

**Fig. 6:**
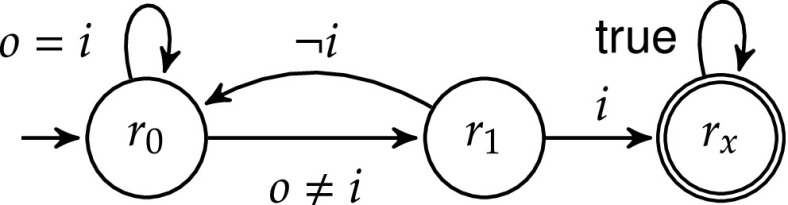
The safety automaton $\varphi$

#### Example 1

Consider the safety automaton $\mathcal {\varphi }$ in Fig. 6, where *i* is an input, *o* is an output, and $r_x$ is an unsafe state. The winning region is $W=\{r_0\}$ because from $r_1$ the input *i* controls whether $r_x$ is visited. The shield must be allowed to deviate from the original transition $r_0\rightarrow r_1$ if $o\ne i$. In $r_1$ it is too late because avoiding an unsafe state can no longer be guaranteed, given that the shield can modify the value of *o* but not *i*.

The second phase (Step 1-b) expands the state space *Q* to $2^{Q}$ via a subset construction with the following rationale. If $\mathcal {D}$ makes a mistake (i.e., picks outputs such that $\varphi$ enters a state $q\not \in W$), the shield has to “guess” what the design actually meant to do. $\mathcal {U}$ considers all output letters that would have avoided leaving *W* and continues monitoring $\mathcal {D}$ from all the corresponding successor states in parallel. Thus, $\mathcal {U}$ is a subset construction of $\varphi$, where a state $u\in U$ of $\mathcal {U}$ represents a set of states in $\varphi$.

The third phase (Step 1-c) expands the state space of $\mathcal {U}$ by adding a Boolean variable *d* to indicate whether the shield is in the recovery period, and a Boolean output variable *z*. Initially *d* is $\bot$. Whenever there is a property violation by $\mathcal {D}$, *d* is set to $\top$ in the next step. If $d=\top$, the shield is in the recovery phase and can deviate. In order to decide when to set *d* from $\top$ to $\bot$, we add an output *z* to the shield. If $z = \top$ and $d = \top$, then *d* is set to $\bot$.

From $\varphi$, the final violation monitor is $\mathcal {U} = (U, u_0, \varSigma ^u, \delta ^u)$, with the states $U = (2^{Q} \times \{\top , \bot \})$, the initial state $u_0 = (\{q_0\}, \bot )$, the input/output alphabet $\varSigma ^u = \varSigma _I\times \varSigma _O^u$ with $\varSigma _O^u = 2^{O \cup z},~$ and the next-state function $\delta ^u$ which obeys the following rules:

1. $$\delta ^u((u,d), ({\sigma _I}, {\sigma _O})) = \bigl (\{q' \!\in \!W \mid \exists q\in u, {\sigma _O}' \in \varSigma _O^u {{\mathrm{\mathbin {.}}}}\delta (q,({\sigma _I},{\sigma _O}')) = q'\}, \top \bigr )$$ if $$\forall q \in u {{\mathrm{\mathbin {.}}}}\delta (q,({\sigma _I}, {\sigma _O})) \not \in W$$, and
2. $\delta ^u((u,d), \sigma ) = \bigl (\{q'\!\in \!W \mid \exists q\!\in \!u {{\mathrm{\mathbin {.}}}}\delta (q,\sigma ) = q'\}, \texttt {dec}(d)\bigr )$ if $\exists q \!\in \!u {{\mathrm{\mathbin {.}}}}\delta (q,\sigma ) \!\in \!W$, and $\texttt {dec}(\bot ) = \bot$, and if *z* is $\top$ then $\texttt {dec}(\top ) = \bot$, else $\texttt {dec}(\top ) = \top$.

Our construction sets $d=\top$ whenever $\mathcal {D}$ leaves the winning region, rather than waiting until the design enters an unsafe state. Conceptually, this allows $\mathcal {S}$ to take remedial action as soon as the “the crime is committed” but before the damage actually takes place, which may be too late to correct erroneous outputs of the design.

#### Example 2

We illustrate the construction of $\mathcal {U}$ using the specification $\varphi$ from Fig. 2, which becomes a safety automaton if we make all missing edges point to an (additional) unsafe state. The winning region consists of all safe states, i.e., $W = \{F,N,S\}$. The resulting violation monitor $\mathcal {U}$ is illustrated in Fig. 8. In this example, *z* is always set to $\top$. For the sake of clarity, *z* is not shown. The update of *d* is as follows: Whenever the design commits a violation (indicated by red dashed edges), then *d* is set to $\top$. Otherwise, *d* is set to $\bot$.

Let us take a closer look at some of the edges of $\mathcal {U}$ in Fig. 8. If the current state is $(\{F\},\bot )$ and $\mathcal {U}$ observes the output *gg* from the design, a specification violation occurs. We assume that $\mathcal {D}$ meant to give an allowed output, i.e., either *gr* or *rr*. $\mathcal {U}$ continues to monitor both *F* and *N*; thus, $\mathcal {U}$ enters the state $(\{F,N\},\top )$. If the next observation is again *gg*, which is neither allowed in *F* nor in *N*, we know that a second violation occurred. $\mathcal {U}$ continues to monitor the design from all states that are reachable from the current set of monitored states: in this case all three states and $\mathcal {U}$ enters the state $(\{F,N,S\},\top )$. If the next observation is *rr*, then *d* is set to $\bot$ and $\mathcal {U}$ enters the state $(\{F\},\bot )$. This constitutes the end of the recovery period

**Fig. 7:**
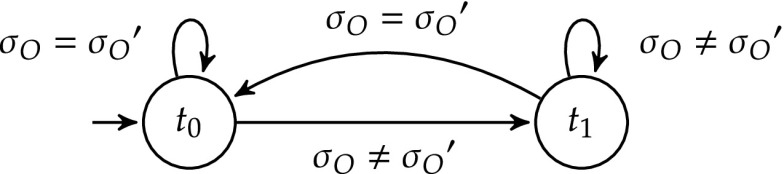
The deviation monitor $\mathcal {T}$

*Step 2 Constructing the deviation monitor* $\mathcal {T}$: We build $\mathcal {T} = (T, t_0, \varSigma _O\times \varSigma _O, \delta ^t)$ to monitor deviations between the shield’s and design’s outputs. Here, $T = \{t_0, t_1\}$ and $\delta ^t(t, ({\sigma _O}, {\sigma _O}')) = t_0$ iff ${\sigma _O}= {\sigma _O}'$. That is, if there is a deviation in the current time step, then $\mathcal {T}$ will be in $t_1$ in the next time step. Otherwise, it will be in $t_0$. This deviation monitor is shown in Fig. 7.

**Fig. 8:**
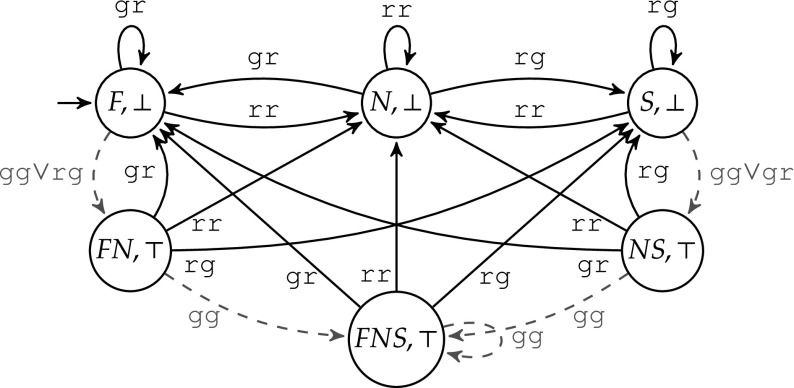
Violation monitor $\mathcal {U}$ of Example 2

*Step 3 Constructing and solving the safety game* $\mathcal {G}^s$. We construct a safety game $\mathcal {G}^s$ such that any shield that implements a winning strategy for $\mathcal {G}^s$ is allowed to deviate in the recovery period only, and the output of the shield is always correct.

Let the automata $\mathcal {U}$ and $\mathcal {T}$ and the safety automaton $\varphi$ be given. Let $W\subseteq F$ be the winning region of $\varphi$ when considered as a safety game. We construct a safety game $\mathcal {G}^s = (G^s, g_0^s, \varSigma _I^s, \varSigma _O^s$ $\delta ^s, F^s)$, which is the synchronous product of $\mathcal {U}$, $\mathcal {T}$ and $\varphi$ such that $G^s= U \times T \times Q$ is the state space, $g_0^s = (u_0, t_0, q_0)$ is the initial state, $\varSigma _I^s = \varSigma _I\times \varSigma _O$ is the input alphabet, $\varSigma _O^s = \varSigma _O$ is the output alphabet, $\delta ^s$ is the next-state function, and $F^s$ is the set of safe states, such that $\delta ^s\bigl ((u, t, q), ({\sigma _I}, {\sigma _O}), {\sigma _O}'\bigr ) =$

$$\begin{aligned} \bigl ( \delta ^u(u,({\sigma _I}, {\sigma _O})), \delta ^t(t,({\sigma _O}, {\sigma _O}')), \delta (q, ({\sigma _I}, {\sigma _O}')) \bigr ), \end{aligned}$$

and $$F^s = \{(u, t, q)\in G^s \mid ((q \in W) \wedge (u=(w \in 2^W,\bot ) \rightarrow t=t_0))\}$$.

In the definition of $F^s$, we require that $q \in W$, i.e., it is a state of the winning region, which ensures that the shield output will satisfy $\varphi$. The second term ensures that the shield can only deviate in the recovery period, i.e., while $d = \top$ in $\mathcal {U}$.

We use standard algorithms for safety games (cf. [19]) to compute the winning region $W^s$ and the permissive winning strategy $\rho _s: G\times \varSigma _I\rightarrow 2^{\varSigma _O}$ that is not only winning for the system, but also subsumes all memoryless winning strategies.

*Step 4 Constructing the Büchi game* $\mathcal {G}^b$. A shield $\mathcal {S}$ that implements the winning strategy $\rho _s$ of the safety game ensures correctness ($\mathcal {D}\circ \mathcal {S}\models \varphi$) and keeps the output of the design $\mathcal {D}$ intact if $\mathcal {D}$ does not violate $\varphi$. What is still missing is to keep the number of deviations per violation to a minimum. As a basic requirement, we would like the recovery period to be over infinitely often. We will see later (in Theorem 1) that this basic requirement is enough to ensure not only a finite recovery period but also the shortest possible recovery period. This requirement can be formalized as a Büchi winning condition. We construct the Büchi game $\mathcal {G}^b$ by applying the permissive safety strategy $\rho ^s$ to the game graph $\mathcal {G}^s$.

Given the safety game $\mathcal {G}^s=(G^s, g_0^s, \varSigma _I^s, \varSigma _O^s, \delta ^s, F^s)$ with the non-deterministic winning strategy $\rho ^s$ and the winning region $W^s$, we construct a Büchi game $\mathcal {G}^b=(G^b, g_0^b, \varSigma _I^b, \varSigma _O^b, \delta ^b, F^b)$ such that $G^b=W^s$ is the state space, the initial state $g_0^b=g_0^s$ and the input/output alphabet $\varSigma _I^b=\varSigma _I^s$ and $\varSigma _O^b=\varSigma _O^s$ remain unchanged, $\delta ^b=\delta ^s\cap \rho ^s$ is the transition function, and $F^b = \{(u, t, q)\in W^s \mid u=(w \in 2^W,\bot )\}$ is the set of accepting states. A play is winning if $d=\bot$ infinitely often.

*Step 5 Solving the Büchi game* $\mathcal {G}^b$. We use standard algorithms for Büchi games (cf. e.g. [30]) to compute a winning strategy $\rho ^b$ for $\mathcal {G}^b$. If a winning strategy exists, we implement this strategy in a new reactive system $\mathcal {S}= (G^b, g^b_0, \varSigma _I^b, \varSigma _O^b, \delta ', \rho ^b)$ with $\delta '(g,{\sigma _I}) = \delta ^b(g,{\sigma _I},\rho ^b(g,{\sigma _I}))$.

#### Theorem 1

A system that implements the winning strategy $\rho ^b$ in the Büchi game $\mathcal {G}^b=(G^b, g_0^b, \varSigma _I^b, \varSigma _O^b, \delta ^b, F^b)$ in a new reactive system $\mathcal {S}= (G^b, g^b_0, \varSigma _I^b, \varSigma _O^b, \delta ', \rho ^b)$ with $\delta '(g,{\sigma _I}) = \delta ^b(g,{\sigma _I},\rho ^b(g,{\sigma _I}))$ is a *k*-stabilizing shield for the smallest *k* possible.

#### Proof

Since $\rho ^b \sqsubseteq \rho ^s$, implementing $\rho ^b$ ensures correctness ($\mathcal {D}\circ \mathcal {S}\models \varphi$) and that $\mathcal {S}$ does not deviate from $\mathcal {D}$ unnecessarily. Therefore, $\mathcal {S}$ is a shield (see Definition 1). Furthermore, the strategy $\rho ^b$ ensures that the recovery period is over infinitely often. Since winning strategies for Büchi games are subgame optimal, a shield that implements $\rho ^b$ ends deviations at any state after the smallest number of steps possible, i.e., $\mathcal {S}$ adversely *k*-stabilizes any trace for the smallest *k* possible. Hence, $\mathcal {S}$ is a *k*-stabilizing shield (see Definition 3). $\square$

The standard algorithm for solving Büchi games contains the computation of attractors. The *i*-th attractor for the system contains all states from which the system can “force” a visit of an accepting state in *i* steps. For all states $g\in G^b$ of the game $\mathcal {G}^b$, the attractor number *i* of *g* corresponds to the smallest number of steps within which the recovery phase can be guaranteed to end.

#### Theorem 2

Let $\varphi =\{Q, q_{0}, \varSigma , \delta , F\}$ be a safety specification and |*Q*| be the cardinality of the state space of $\varphi$. A *k*-stabilizing shield with respect to $\varphi$ can be synthesized in $\mathcal {O}(|Q|^3 2^{|Q|})$ time if it exists.

#### Proof

Our safety game $\mathcal {G}^s$ and our Büchi game $\mathcal {G}^b$ have at most $m=(2 \cdot 2^{|Q|})\cdot 2 \cdot |Q|$ states and at most $n=m^2$ edges. Safety games can be solved in $\mathcal {O}(m+n)$ time and Büchi games in $\mathcal {O}(m\cdot n)$ time [30]. $\square$

While an exponential runtime may not look appealing at the first glance, keep in mind that the critical safety properties of a system are typically simple and that the complexity of the design is irrelevant for the shield synthesis procedure.

### Admissible shields

In this section we define admissible shields. We distinguish between two situations. In states of the design in which a finite number *k* of deviations can be guaranteed, an admissible shield takes an adversarial view on the design: it guarantees recovery within *k* steps regardless of system behavior, for the smallest *k* possible. In these states, the strategy of an admissible shield conforms to the strategy of a *k*-stabilizing shield. In all other states, admissible shields take a collaborative view: the admissible shield will attempt to work with the design to recover as soon as possible. In particular, an admissible shield plays an admissible strategy, that is, a strategy that cannot be beaten in recovery speed if the design acts cooperatively. We will now define admissible shields.

#### Definition 4

*(Adversely subgame optimal shield)* A shield $\mathcal {S}$ is *adversely subgame optimal* if, for any trace $\overline{\sigma }\in \varSigma ^*$, $\mathcal {S}$ adversely $k-$stabilizes $\overline{\sigma }$ (Definition 2) and there exists no shield that adversely *l*-stabilizes $\overline{\sigma }$ for any $l<k$.

An adversely subgame optimal shield $\mathcal {S}$ guarantees to deviate in response to an error for at most *k* time steps, for the smallest *k* possible.

#### Definition 5

Let $\varphi$ be a safety specification and let $\overline{\sigma }= ({\overline{\sigma _I}}||{\overline{\sigma _O}}) \in \varSigma ^{\omega }$ be a correct trace. Let $\overline{\sigma }^f = ({\overline{\sigma _I}}||{\overline{\sigma _O}}^f)\in \varSigma ^{\omega }$ be a trace in which $\forall i$ with ${\overline{\sigma _O}}[i]\ne {\overline{\sigma _O}}^f[i]$ it holds that $\overline{\sigma }[0\dots i-1] \cdot ({\overline{\sigma _I}}[i], {\overline{\sigma _O}}[i]^f)$ is wrong and let $E=\{i \mid {\overline{\sigma _O}}[i] \ne {\overline{\sigma _O}}^f[i]\}$. A shield $\mathcal {S}$ *collaboratively k-stabilizes* $\overline{\sigma }$ if for any wrong output ${\overline{\sigma _O}}^f[i]$ with $i \in E$ the following holds: For any correct output ${\sigma _O}^c\in \varSigma _O$ (i.e., $\overline{\sigma }[0\dots i-1] \cdot ({\overline{\sigma _I}}[i], {\sigma _O}^c)$ is correct), *there exists* a correct trace $({\overline{\sigma _I}}^c||{\overline{\sigma _O}}^c)\in \varSigma ^{\omega }$ (i.e., $\overline{\sigma }[0\dots i-1] \cdot ({\overline{\sigma _I}}[i],{\sigma _O}^c) \cdot ({\overline{\sigma _I}}^c|| {\overline{\sigma _O}}^c)$ is correct), such that

$$\begin{aligned}&\overline{\sigma }^\# := \overline{\sigma }[0 \dots i]^f \cdot ({\overline{\sigma _I}}^c|| {\overline{\sigma _O}}^c) \text {,~} {\overline{\sigma _O}}^\star := \mathcal {S}(\overline{\sigma }^\#),\\&({\overline{\sigma _I}}||{\overline{\sigma _O}}^\star )\models \varphi \text {~and~} \forall j\ge i {{\mathrm{\mathbin {.}}}}{\overline{\sigma _O}}^\star [j] \ne {\overline{\sigma _O}}^\#[j] \rightarrow j-i \le k. \end{aligned}$$

The trace $\overline{\sigma }^f$ results from substituting outputs in $\overline{\sigma }$ by wrong outputs, and *E* contains the indices of the wrong outputs as defined in Sect. 3.3. The shield has to correct any wrong output ${\overline{\sigma _O}}^f[i]$ with $i \in E$ with an output such that, for *some* correct output ${\sigma _O}^c$ and *some* correct continuation $({\overline{\sigma _I}}^c || {\overline{\sigma _O}}^c)$, the shield is able to end the deviation after *k*-steps, and the shielded trace satisfies $\varphi$.

#### Definition 6

(*Collaborative k-Stabilizing Shield*) A shield $\mathcal {S}$ is collaboratively *k*-stabilizing if it collaboratively *k*-stabilizes any finite trace.

A collaborative *k*-stabilizing shield requires that it must be possible to end deviations after *k* steps, for some future input and output of $\mathcal {D}$. It is not necessary that this is possible for all future behavior of $\mathcal {D}$ allowing infinitely long deviations.

#### Definition 7

*(Collaborative subgame optimal shield)* A shield $\mathcal {S}$ is *collaborative subgame optimal* if, for any trace $\overline{\sigma }\in \varSigma ^*$, $\mathcal {S}$ collaboratively $k-$stabilizes $\overline{\sigma }$ and there exists no shield that adversely *l*-stabilizes $\overline{\sigma }$ for any $l<k$.

#### Definition 8

*(Admissible Shield)* A shield $\mathcal {S}$ is admissible if, for any trace $\overline{\sigma }$, whenever there exists a *k* and a shield $\mathcal {S}'$ such that $\mathcal {S}'$ adversely *k*-stabilizes $\overline{\sigma }$, then $\mathcal {S}$ is an adversely subgame optimal shield and adversely *k*-stabilizes $\overline{\sigma }$ for a minimal *k*. If such a *k* does not exist for trace $\overline{\sigma }$, then $\mathcal {S}$ acts as a collaborative subgame optimal shield and collaboratively *k*-stabilizes $\overline{\sigma }$ for a minimal *k*.

An admissible shield ends deviations as soon as possible. In all states of the design $\mathcal {D}$ from which it is possible to *k*-adversely stabilize traces, an admissible shield does this for the smallest *k* possible. In all other states, the shield corrects the output in such a way that there exists design’s inputs and outputs such that deviations end after *l* steps, for the smallest *l* possible.

### Synthesizing admissible shields

The flow of the synthesis procedure for admissible shields is similar to the flow for synthesizing *k*-stabilizing shields, and is illustrated in Fig. 5. In order to synthesize an admissible shield, a Büchi game $\mathcal {G} ^b$ is constructed in the same way as for *k*-stabilizing shields. The difference lies in the computation of the strategy of the Büchi game $\mathcal {G} ^b$: for *k*-stabilizing shields we compute a winning strategy of $\mathcal {G} ^b$, and for admissible shields we compute an admissible strategy. Given is a safety specification $\varphi = \{\varphi _1,\ldots ,\varphi _l\} = (Q, q_{0}, \varSigma _I\times \varSigma _O, \delta ,F)$. Starting from $\varphi$, our shield synthesis procedure is as follows:

*Steps 1–4* Perform as in Sect. 3.4.

*Step 5 Solving the Büchi game* $\mathcal {G}^b$. A Büchi game $\mathcal {G}^b$ may contain reachable states for which $d=\bot$ cannot be enforced infinitely often, i.e., states from which a recovery in a finite time cannot be guaranteed. We implement an admissible strategy that visits states with $d=\bot$ infinitely often whenever possible. This criterion essentially asks for a strategy that is winning with the help of the design.

The admissible strategy $\rho ^b$ for a Büchi game $\mathcal {G}^b=(G^b, g_0^b, \varSigma _I^b, \varSigma _O^b, \delta ^b, F^b)$ can be computed as follows[19]:

1. Compute the winning region $W^b$ and a winning strategy $\rho _w^b$ for $\mathcal {G}^b$ (cf. [30]).
2. Remove all transitions that start in $W^b$ and do not belong to $\rho _w^b$ from $\mathcal {G}^b$. This results in a new Büchi game $\mathcal {G}_1^b=(G^b, g_0^b, \varSigma _I^b, \varSigma _O^b, \delta _1^b, F^b)$ with $(g,({\sigma _I},{\sigma _O}),g')\in \delta _1^b$ if $(g,{\sigma _I}, {\sigma _O})\in \rho _w^b$ or if $\forall {\sigma _O}' \in \varSigma _O^b {{\mathrm{\mathbin {.}}}}(g,{\sigma _I}, {\sigma _O}')\notin \rho _w^b \wedge (g,({\sigma _I},{\sigma _O}),g')\in \delta ^b$.
3. In the resulting game $\mathcal {G}_1^b$, compute a cooperatively winning strategy $\rho ^b$. In order to compute $\rho ^b$, one first has to transform all input variables to output variables. This results in the Büchi game $\mathcal {G}_2^b=(G^b, g_0^b, \emptyset , \varSigma _I^b\times \varSigma _O^b, \delta _1^b, F^b)$. Afterwards, $\rho ^b$ can be computed with the standard algorithm for the winning strategy on $\mathcal {G}_2^b$.

The strategy $\rho ^b$ is an admissible strategy of the game $\mathcal {G}^b$, since it is winning and cooperatively winning [19]. Whenever the game $\mathcal {G}^b$ starts in a state of the winning region $W^b$, any play created by $\rho _w^b$ is winning. Since $\rho ^b$ coincides with $\rho _w^b$ in all states of the winning region $W^b$, $\rho ^b$ is winning. We know that $\rho ^b$ is cooperatively winning in the game $\mathcal {G}_1^b$. A proof that $\rho ^b$ is also cooperatively winning in the original game $\mathcal {G}^b$ can be found in [19].

#### Theorem 3

A shield that implements the admissible strategy $\rho ^b$ in the Büchi game $\mathcal {G}^b=(G^b, g_0^b, \varSigma _I^b, \varSigma _O^b, \delta ^b, F^b)$ in a new reactive system $\mathcal {S}= (G^b, g^b_0, \varSigma _I^b, \varSigma _O^b, \delta ', \rho ^b)$ with $\delta '(g,{\sigma _I}) = \delta ^b(g,{\sigma _I},\rho ^b(g,{\sigma _I}))$ is an admissible shield.

#### Proof

Since $\rho ^b \sqsubseteq \rho ^s$, $\mathcal {S}$ is a shield according to Definition 1. $\mathcal {S}$ is an adversely subgame optimal shield (see Definition 4) for all states of the design in which a finite number of deviations can be guaranteed, since $\rho ^b$ is winning for all winning states of the Büchi game $\mathcal {G}^b$, and winning strategies for Büchi games are subgame optimal. Furthermore, $\mathcal {S}$ is a collaborative subgame optimal shield (see Definition 7), since $\rho ^b$ is cooperatively winning in the Büchi game $\mathcal {G}^b$ and cooperative winning strategies for Büchi games are subgame optimal for some inputs. Therefore, $\mathcal {S}$ is an admissible shield (see Definition 8). $\square$

### Shields with a fail-safe mode

When a property violation becomes unavoidable, *k*-stabilizing shields and admissible shields enter a recovery phase, where the shield is allowed to deviate. In the case that it can be assumed that specification violations are rare events, the construction of *k*-stabilizing shields and admissible shields can be modified to tolerate the next violation only after the recovery phase. If a second violation happens within the recovery period, the shield enters a fail-safe mode, where it enforces the specification, but stops minimizing the deviations from that point on, i.e., it loses the ability to recover.

For many practical examples, this modification speeds up the synthesis process and results in significantly smaller implementations of $\mathcal {S}$. This is because the set of states to be monitored by $\mathcal {U}$ generally becomes smaller. In the case of a violation, a shield enters its recovery phase and $\mathcal {U}$ observes $\mathcal {D}$ from all states that $\mathcal {D}$ could reach under the current input and a correct output. Consider the case of a second violation within the recovery phase. For shields without a fail-safe mode, $\mathcal {U}$ has to monitor the set of all input-enabled states that are reachable from the current set of monitored states. For shields with a fail-safe mode, $\mathcal {U}$ just enters a single special fail-safe state.

#### Construction of shields with a fail-safe mode

In order to construct a *k*-stabilizing shield or an admissible shield with a fail-safe mode, only small changes in the construction of the violation monitor and the Büchi game are necessary.

*Changes in Step 1 Constructing the violation monitor* $\mathcal {U}$: Only the third phase of the construction of $\mathcal {U}$ (see Sect. 3.4, Step 1-c) needs to be modified.

As before, we expand the state space of $\mathcal {U}$ by adding a variable *d* to indicate whether the shield is in the recovery phase. Additionally, we now add a special fail-safe state $u_E$ to indicate whether the shield is in the fail-safe mode. If a second violation happens while $d=\top$, then the shield enters the fail-safe mode.

From $\varphi$, the final violation monitor is $\mathcal {U} = (U, u_0, \varSigma ^u, \delta ^u)$, with the set of states $U = (2^{Q}\cup u_E) \times \{\top , \bot \}$, $u_0 = (\{q_0\}, \bot )$, the input/output alphabet $\varSigma ^u = \varSigma _I\times \varSigma _O^u$ with $\varSigma _O^u = 2 ^ {O \cup z}$, and the next-state function $\delta ^u$ that obeys the following rules:

1. $\delta ^u((u_E,\top ), \sigma ) = (u_E,\top )$ (meaning that $u_E$ is a trap state),
2. $$\delta ^u((u,\top ), \sigma ) = (u_E,\top )$$ if $$\forall q \in u {{\mathrm{\mathbin {.}}}}\delta (q,\sigma ) \not \in W$$,
3. $$\delta ^u((u,\bot ), ({\sigma _I}, {\sigma _O})) = \bigl (\{q' \!\in \!W \mid \exists q\in u, {\sigma _O}' \in \varSigma _O^u {{\mathrm{\mathbin {.}}}}\delta (q,({\sigma _I},{\sigma _O}')) = q'\}, \top \bigr )$$ if $$\forall q \in u {{\mathrm{\mathbin {.}}}}\delta (q,({\sigma _I}, {\sigma _O})) \not \in W$$, and
4. $\delta ^u((u,d), \sigma ) = \bigl (\{q'\!\in \!W \mid \exists q\!\in \!u {{\mathrm{\mathbin {.}}}}\delta (q,\sigma ) = q'\}, \texttt {dec}(d)\bigr )$ if $\exists q \!\in \!u {{\mathrm{\mathbin {.}}}}\delta (q,\sigma ) \!\in \!W$, and $\texttt {dec}(\bot ) = \bot$, and if *z* is $\top$ then $\texttt {dec}(\top ) = \bot$, else $\texttt {dec}(\top ) = \top$.

##### Example 3

**Fig. 9:**
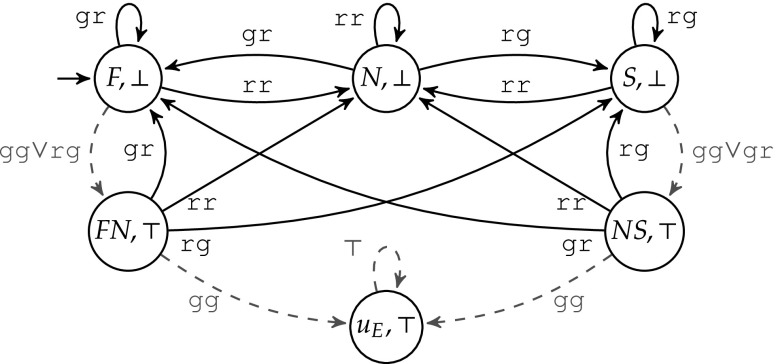
Violation monitor $\mathcal {U}$ of Example 2 with a fail-safe mode

Figure 9 shows $\mathcal {U}$ with a fail-safe mode using the specification $\varphi$ from Fig. 2. If $d=\top$ at the violation, the next state is $u_E$. Otherwise, *d* is set to $\bot$.

*Changes in Step 4 Constructing the Büchi game* $\mathcal {G}^b$: We change the original construction of the Büchi game of Sect. 3.4 only slightly by adding the state $(u_E, \top )$ to the set of accepting states. The intuition is that, if the shield enters the state $(u_E, \top )$, it can stop minimizing the deviations, and the Büchi condition is trivially satisfied.

### Liveness-preserving shields

Reactive systems usually not only satisfy safety properties, but are also expected to satisfy liveness properties [2], which guarantee that certain good events eventually happen. Unfortunately, it is not guaranteed that the corrections of the shield preserve the liveness properties satisfied by the design (without shielding).

#### Definition 9

*(Liveness-Preserving Shield)* A shield preserves a given set of liveness properties if any liveness properties satisfied by the design without shielding are also satisfied by the shield.

We now give a example where $\mathcal {S}$ destroys a liveness property satisfied by $\mathcal {D}$.

#### Example 4

Consider a simple arbiter, with one input signal *r*, with which clients request permissions, and two output signals $a_1$ and $a_2$ to grant resource 1 and resource 2. The implementation of the arbiter is already given, but the full specification of the arbiter is unknown. Suppose we know that the arbiter satisfies the following two liveness properties:

- $\varphi ^l_{1}$: Resource 1 has to be granted infinitely often, i.e., ${{\mathrm{\mathsf {G}}}}{{\mathrm{\mathsf {F}}}}(a_1)$ in LTL [32].
- $\varphi ^l_{2}$: Resource 2 has to be granted infinitely often, i.e., ${{\mathrm{\mathsf {G}}}}{{\mathrm{\mathsf {F}}}}(a_2)$ in LTL.

**Fig. 10:**
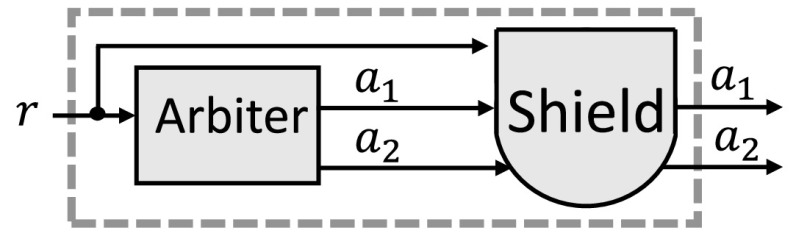
Attaching the shield (Example 4)

**Fig. 11:**
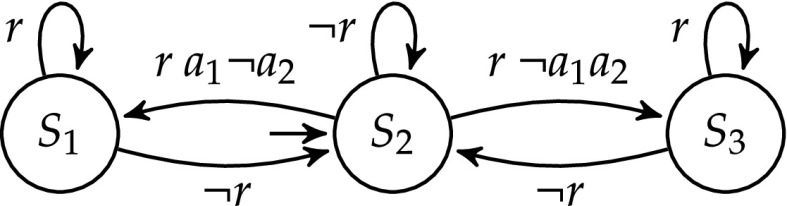
Safety specification $\varphi ^s$ (Example 4)

We attach a shield to the arbiter as shown in Fig. 10 to enforce the safety property $\varphi ^s$ expressed by the safety automaton in Fig. 11. Although this safety automaton is not a complete specification of the arbiter, the corresponding shield can enforce important properties of the arbiter. $\varphi ^s$ states that if there is a request *r* in state $S_2$, then one resource has to be granted immediately: either resource 1 with $a_1\lnot a_2$, or resource 2 with $\lnot a_1 a_2$. There is also an error state, which is not shown. Missing edges lead to this error state, e.g., granting both resources (with $a_1 a_2$) or no resource (with $\lnot a_1 \lnot a_2$) after a request *r* in state $S_2$ is never allowed.

**Table 3:** Shield $\mathcal {S}$ correcting the arbiter (Example 4)

Time step	1	2	3	4	5
Arbiter	$\lnot r \lnot a_1 \lnot a_2$	${{\varvec{r}}}~{{\varvec{a}}}_{\mathbf{1}} {{\varvec{a}}}_{\mathbf{2}}$	$\lnot r \lnot a_1 \lnot a_2$	${{\varvec{r}}}~{{\varvec{a}}}_{\mathbf{1}} {{\varvec{a}}}_{\mathbf{2}}$	$\dots$
Shield	$\lnot r \lnot a_1 \lnot a_2$	${\textit{r}} ~ \lnot {\textit{a}}_{1}{\textit{a}}_{2}$	$\lnot r \lnot a_1 \lnot a_2$	${\textit{r}} ~ \lnot {\textit{a}}_{1} {\textit{a}}_{2}$	$\dots$

Table 3 shows how $\mathcal {S}$ may correct the arbiter. In step 1, we are $S_2$ and the arbiter receives no request (i.e., $\lnot r$). In this case, every possible output from the arbiter is accepted by the shield. In step 2, the arbiter receives a request (i.e., *r*), but grants both resources at once (i.e., $a_1 a_2$), which violates $\varphi ^s$. The shield corrects the output to $\lnot a_1 a_2$. In step 3, the arbiter receives $\lnot r$ , and sets all outputs to $\bot$. The shield accepts the output, and ends the deviation. From there on, everything repeats infinitely often.

Let us analyse the corrections of the shield with respect to the liveness properties $\varphi ^l_{1}$ and $\varphi ^l_{2}$. The arbiter gave $a_1$ and $a_2$ infinitely often, thereby satisfying both $\varphi ^l_{1}$ and $\varphi ^l_{2}$. The output of the shield however never included the symbol $a_1$. Although the arbiter satisfied all liveness properties, through the correction of the shield, the first liveness property $\varphi ^l_{1}$ is violated.

Next, we discuss an extension to the *k*-stabilizing and the admissible shield synthesis procedure that allows liveness-preserving shielding.

#### Synthesizing liveness-preserving shields

To construct a system that has all the properties of a shield (*k*-stabilizing or admissible) and is liveness-preserving, we create and solve a Streett game with two pairs. The first Streett pair, called the *shielding pair*, encodes that the recovery phase has to end infinitely often. The second Streett pair, called the *liveness-preservation pair*, encodes that if the design satisfies all liveness properties, then the shield has to preserve all liveness properties.

Let $\varphi ^s = \{\varphi _1^s,\ldots ,\varphi _m^s\} = (Q^s, q^s_{0}, \varSigma ^s, \delta ^s, F^s)$ be the safety specification to be enforced by the shield. Let $\varphi ^l = \{\varphi _1^l,\ldots ,\varphi _n^l\}$ be the set of liveness properties that if satisfied by the design, have to be preserved after shielding. Each $\varphi _i^l$ is represented as a Büchi automaton $\varphi _i^l = (Q_i^l, q_{0,i}^l, \varSigma ^l, \delta _i^l, F^l_i)$, with $F^l_i$ the set of states that have to be visited infinitely often. The synchronous product $\varphi ^l$ of $\{\varphi _1^l,\ldots ,\varphi _n^l\}$ defines an automaton with generalized Büchi acceptance condition $\varphi ^l = (Q^l, q_{0}^l, \varSigma ^l, \delta ^l, \{F^l_1,\dots ,F^l_n\})$. Given $\varphi ^s$ and $\varphi ^l$, our shield synthesis procedure consists of three steps.

*Step 1 Encode the shielding Streett pair* $\mathbf {\langle E_1, F_1 \rangle }$. Given the safety specification $\varphi ^s$, we construct a one-pair Streett game $\mathcal {G}^{s1}$. In the case of *k*-stabilizing shields, we construct $\mathcal {G}^{s1}$ in such a way that the winning strategy corresponds to a *k*-stabilizing shield. In case of admissible shields, we construct $\mathcal {G}^{s1}$ such that the admissible strategy implements an admissible shield.

First (Step 1-a), we construct a Büchi game $\mathcal {G}^b = (G^b, g^b_0, \varSigma _I\times \varSigma _O, \varSigma _O, \delta ^b, F^b)$, using the construction described in Sect. 3.4 for *k*-stabilizing shields, or the construction of Sect. 3.6 for admissible shields. In both cases, the transition relation $\delta ^b$ contains only transitions in which the output of the shield satisfies $\varphi ^s$ and there are no illegal deviations between the output of the shield and the output of the design. $F^b$ covers all states with $d=\bot$; i.e. if these states are visited infinitely often, the recovery phase will be over infinitely often.

Next (Step 1-b), we transform the Büchi game $\mathcal {G}^b$ into the Streett game $\mathcal {G}^{s1}=(G^{s1}, g^{s1}_0, \varSigma _I\times \varSigma _O, \varSigma _O, \delta ^{s1}, \langle E_1, F_1 \rangle )$ such that $G^{s1}=G^b$, $g^{s1}_0=g^b_0$, $\delta ^{s1}=\delta ^b$ and $\langle E_1, F_1 \rangle =\langle G^{s1}, F^b \rangle$. The intuition is that the states in $F_1=F^b$ must always be visited infinitely often (the deviation phase should end infinitely often); therefore, we set $E_1$ to the set of all states.

*Step 2 Encode the liveness-preservation Streett pair* $\mathbf {\langle E_2, F_2 \rangle }$. From the liveness specification $\varphi ^l = (Q^l, q_{0}^l, \varSigma ^l, \delta ^l, \{F^l_1,\dots ,F^l_n\})$, we construct another one-pair Streett game $\mathcal {G}^{s2}$ such that a winning strategy in this game corresponds to a liveness-preserving implementation. Therefore, we turn the condition that if the design satisfies $\varphi ^l$, then the shield has to satisfy $\varphi ^l$ as well, into the Liveness-Preservation Streett pair $\mathbf {\langle E_2, F_2 \rangle }$. The construction of $\mathcal {G}^{s2}$ consists of two steps.

In the first phase (Step 2-a), we create a GR(1) game $\mathcal {G}^g=(G^g, g^g_0, \varSigma _I\times \varSigma _O, \varSigma _O, \delta ^g, Acc)$, such that $G^g= Q^l \times Q^l$ is the state space, $g_0 = (q^l_0, q^l_0)$ is the initial state, $\varSigma _I\times \varSigma _O$ is the input, $\varSigma _O$ is the output, $\delta ^g$ is the next-state function, and *Acc* is the GR(1) acceptance condition such that $\delta ^g\bigl ((q, q'), ({\sigma _I}, {\sigma _O}), {\sigma _O}'\bigr ) =$

$$\begin{aligned} \bigl ( \delta ^l(q,({\sigma _I}, {\sigma _O})), \delta ^l(q', ({\sigma _I}, {\sigma _O}')) \bigr ), \end{aligned}$$

and $$Acc = \bigwedge _i \inf (\overline{q}) \wedge F_i^l \ne \emptyset \rightarrow \bigwedge _i \inf (\overline{q'}) \wedge F_i^l \ne \emptyset $$ with $$\overline{g^g}=\overline{q}||\overline{q'}= (q_0 q'_0)(q_1 q'_1)\cdots $$.

In the second phase (Step 2-b), the GR(1) game $\mathcal {G}^g$ is transformed into a one-pair Streett game $\mathcal {G}^{s2}=(G^{s2}, g^{s2}_0, \varSigma _I\times \varSigma _O, \varSigma _O, \delta ^{s2}, \langle E_2, F_2 \rangle )$ via a counting construction [5].

*Step 3: Construct and solve the two-pair Streett game* From $\mathcal {G}^{s1}$ and $\mathcal {G}^{s2}$, we construct a Streett game $\mathcal {G}^{st}$ with two Streett pairs: $\mathcal {G}^{st} = (G^{st}, g^{st}_0, \varSigma _I\times \varSigma _O, \varSigma _O, \delta ^{st}, Acc)$ with the state space $G^{st}=G^{s1}\times G^{s2}$, the initial state $g^{st}_0=(g^{s1}_0,g^{s2}_0)$, the next-state function $\delta ^{st}$ and the acceptance condition *Acc*, such that $\delta ^{st}\bigl ((g^{s1}, g^{s2}), ({\sigma _I}, {\sigma _O}), {\sigma _O}'\bigr ) =$

$$\begin{aligned} \bigl ( \delta ^{s1}(g^{s1},({\sigma _I}, {\sigma _O})), \delta ^{s2}(g^{s2}, ({\sigma _I}, {\sigma _O}')) \bigr ), \end{aligned}$$

and $Acc = \{\langle E_1, F_1\rangle , \langle E_2, F_2\rangle \}$. A winning strategy for the two-pair Streett game $\mathcal {G}^{st}$ corresponds to a liveness-preserving *k*-stabilizing shield. The admissible strategy for $\mathcal {G}^{st}$ implements an admissible shield. Streett games with *n* Streett pairs can be solved using the recursive fixpoint algorithm of [31].

### Experimental results

We implemented our *k*-stabilizing and admissible shield synthesis procedures in a Python tool that takes a set of safety automata defined in a textual representation as input. The first step in our synthesis procedure is to build the product of all safety automata and construct the violation monitor (see Sects. 3.4 and 3.6). This step is performed on an explicit representation. For the remaining steps we use Binary Decision Diagrams (BDDs) [10] for symbolic representation.

We have conducted two sets of experiments, where the benchmarks are (1) selected properties for an ARM AMBA bus arbiter [7], and (2) selected properties from LTL specification patterns [16]. The source code of our proof-of-concept synthesis tool as well as the input files and instructions to reproduce our experiments are available for download^1^. All experiments were performed on a machine with an Intel i5-3320M CPU@2.6 GHz, 8 GB RAM, and a 64-bit Linux.

#### ARM AMBA bus arbiter example

**Fig. 12:**
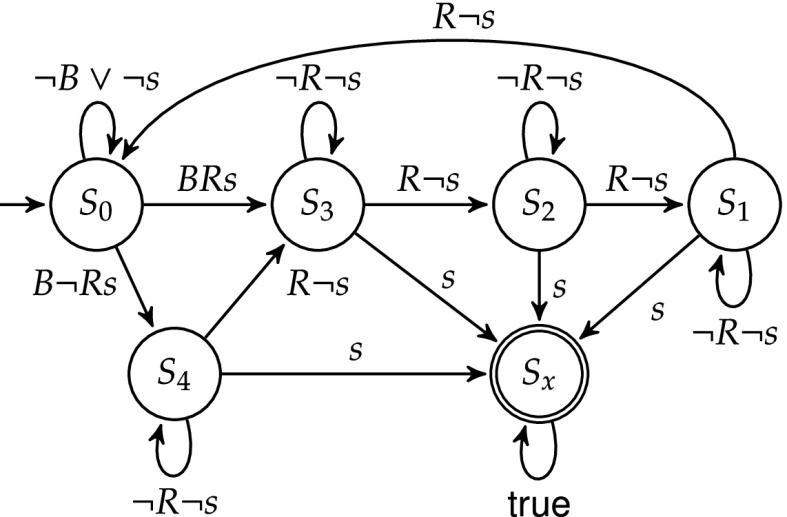
Guarantee 3 from [7]

We used properties of an ARM AMBA bus arbiter [7] as input to our shield synthesis tool. We present the result on one example property, and then present the performance results for other properties. The property that we enforced was Guarantee 3 from the specification of [7], which says that if a length-four locked burst access starts, no other access can start until the end of this burst. The safety automaton is shown in Fig. 12, where *B*, *R*, and *s* are short for $\texttt {hmastlock} \wedge \texttt {HBURST=BURST4}$, HREADY, and start, respectively. Upper case signal names are inputs, and lower-case names are outputs of the arbiter. The state $S_x$ is unsafe. $S_0$ is the idle state waiting for a burst to start ($B \wedge s$). The burst is over if input *R* has been $\top$ 4 times. State $S_i$, where $i=1,2,3,4$, means that *R* must be $\top$ for *i* more times. The counting includes the time step where the burst starts, i.e., where $S_0$ is left. Outside of $S_0$, *s* is required to be $\bot$.

Our tool generates a 1-stabilizing shield and an admissible shield within a fraction of a second. The 1-stabilizing shield has 8 latches and 142 (2-input) multiplexers, which is then reduced by ABC [9] to 4 latches and 77 AIG gates. The admissible shield has 9 latches and 340 multiplexers, which is reduced by ABC to 7 latches and 271 AIG gates. We verified it against an arbiter implementation for 2 bus masters, where we introduced the following bug: the arbiter does not check *R* when the burst starts, but behaves as if *R* was $\top$. This corresponds to removing the transition from $S_0$ to $S_4$ in Fig. 12, and going to $S_3$ instead. An execution trace is shown in Table 4. The first burst starts with $s=\top$ in Step 3. *R* is $\bot$, so the arbiter counts incorrectly. The erroneous output shows up in Step 7, where the arbiter starts the next burst, which is forbidden, and thus blocked by the shield. The arbiter now thinks that it has started a burst, so it keeps $s=\bot$ until *R* is $\top$ 4 times. In actuality, this burst start has been blocked by the shield, so the shield waits in $S_0$. Only after the suppressed burst is over and the components are in sync again can the next burst can start normally.

**Table 4:** Shield execution results

Step	3	4	5	6	7	8	9	10	11	12
State in Fig. 12	$S_0$	$S_4$	$S_3$	$S_2$	$S_1$	$S_0$	$S_0$	$S_0$	$S_0$	...
State in Arbiter	$S_0$	$S_3$	$S_2$	$S_1$	$S_0$	$S_3$	$S_2$	$S_1$	$S_0$	...
*B*	1	1	1	1	1	1	1	1	1	...
*R*	0	1	1	1	1	1	1	1	1	...
*s* from Arbiter	1	0	0	0	1	0	0	0	0	...
*s* from Shield	1	0	0	0	0	0	0	0	0	...

**Table 5:** Performance for AMBA [7]

Property	|*Q*|	|*I*|	|*O*|	$\mathcal {S}$ with fail-safe mode		$\mathcal {S}$ w/o fail-safe mode	
				*k*	Time (s)	*k*	Time (s)
P1:G1	3	1	1	1	0.1	2	0.1
P2:G1 $+$ 2	5	3	3	1	0.1	2	0.6
P3:G1 $+$ 2 $+$ 3	12	3	3	1	0.1	5	0.26
P4:G1 $+$ 2 $+$ 4	8	3	6	2	7.8	2	12
P5:G1 $+$ 3 $+$ 4	15	3	5	2	65	5	117
P6:G1 $+$ 2 $+$ 3 + 5	18	3	4	2	242	5	325
P7:G1 $+$ 2 $+$ 4 + 5	12	3	7	1	45 (admissible)	2	66 (admissible)
P8:G2 $+$ 3 $+$ 4	17	3	6	2	98 (admissible)	5	129 (admissible)
P9:G1 $+$ 3 $+$ 4 + 5	23	3	6	2	432 (admissible)	5	786 (admissible)

To evaluate the performance of our tool, we ran a stress test with increasingly larger sets of safety properties for the ARM AMBA bus arbiter in [7]. Table 5 summarizes the results. The first columns list the set of specification automata and the number of states, inputs, and outputs of their product automata. The next two columns list the results for shields with a fail-safe mode and the last two columns address shields without a fail-safe mode. In both cases, the table first lists the smallest number of steps under which the shield is able to recover (adversely for the properties P1–P6, cooperatively for properties P7–P9) and second the time for synthesizing a shield in seconds. For the first six properties P1–P6, a finite number *k* of deviations can be guaranteed, and the results for admissible shields conform with the results for *k*-stabilizing shields. For the last 3 experiments P7–P9 no *k*-stabilizing shield exists, and the results are given for admissible shields. Both methods (to create shields with and without a fail safe mode) run sufficiently fast on all properties. In these experiments, having a fail-safe mode does not give a significant speedup to justify the tradeoff of losing the ability to recover for arbitrary failure frequencies.

**Table 6:** Synthesis results for the LTL patterns [16]

No.	Property	*b*	|*Q*|	$\mathcal {S}$ with fail-safe mode			$\mathcal {S}$ w/o fail-safe mode		
				Time (s)	#Lat-ches	#AIG-Gates	Time (s)	#Lat-ches	#AIG-Gates
1	${{\mathrm{\mathsf {G}}}}\lnot p$	–	2	0.01	0	0	0.01	0	0
2	${{\mathrm{\mathsf {F}}}}r \rightarrow (\lnot p \mathbin {\mathsf {U}}r)$	–	4	0.34	2	6	0.01	3	10
3	${{\mathrm{\mathsf {G}}}}(q \rightarrow {{\mathrm{\mathsf {G}}}}(\lnot p))$	–	3	0.34	2	6	0.01	2	8
4	${{\mathrm{\mathsf {G}}}}((q \wedge \lnot r \wedge {{\mathrm{\mathsf {F}}}}r) \rightarrow (\lnot p \mathbin {\mathsf {U}}r))$	–	4	0.34	1	9	0.01	3	15
5	${{\mathrm{\mathsf {G}}}}(q \wedge \lnot r \rightarrow (\lnot p \mathbin {\mathsf {W}}r))$	–	3	0.01	2	14	0.02	3	19
6	${{\mathrm{\mathsf {F}}}}p$	0	3	0.34	1	1	0.01	1	1
6	${{\mathrm{\mathsf {F}}}}p$	256	259	33	18	134	39	18	106
7	$\lnot r \mathbin {\mathsf {W}}(p \wedge \lnot r)$	–	3	0.05	3	11	0.01	5	27
8	${{\mathrm{\mathsf {G}}}}(\lnot q) \vee {{\mathrm{\mathsf {F}}}}(q \wedge {{\mathrm{\mathsf {F}}}}p)$	0	3	0.04	3	11	0.01	4	19
8	${{\mathrm{\mathsf {G}}}}(\lnot q) \vee {{\mathrm{\mathsf {F}}}}(q \wedge {{\mathrm{\mathsf {F}}}}p)$	4	7	0.04	6	79	0.02	6	54
8	${{\mathrm{\mathsf {G}}}}(\lnot q) \vee {{\mathrm{\mathsf {F}}}}(q \wedge {{\mathrm{\mathsf {F}}}}p)$	16	19	0.03	10	162	0.05	10	89
8	${{\mathrm{\mathsf {G}}}}(\lnot q) \vee {{\mathrm{\mathsf {F}}}}(q \wedge {{\mathrm{\mathsf {F}}}}p)$	64	67	0.37	14	349	0.58	14	114
8	${{\mathrm{\mathsf {G}}}}(\lnot q) \vee {{\mathrm{\mathsf {F}}}}(q \wedge {{\mathrm{\mathsf {F}}}}p)$	256	259	34	18	890	38	18	150
9	${{\mathrm{\mathsf {G}}}}(q \wedge \lnot r \rightarrow (\lnot r \mathbin {\mathsf {W}}(p \wedge \lnot r)))$	–	3	0.05	2	12	0.03	5	58
10	${{\mathrm{\mathsf {G}}}}(q \wedge \lnot r \rightarrow (\lnot r \mathbin {\mathsf {U}}(p \wedge \lnot r)))$	12	14	5.4	14	2901	168	16	49840

#### LTL specification patterns

Dwyer et al. [16] studied frequently used LTL specification patterns in verification. As an exercise, we applied our tool to the first 10 properties from their list [1] and summarized the results in Table 6. For a property containing liveness aspects (e.g., something must happen eventually), we imposed a bound on the reaction time to obtain the safety (bounded-liveness) property. The bound on the reaction time is shown in Column 3. The next column lists the number of states in the safety specification. The last columns list the synthesis time in seconds, and the shield size (latches and AIG gates) for *k*-stabilizing shields with and without a fail-safe mode. Overall, both methods run sufficiently fast on all properties and the resulting shield size is small. For certain benchmarks we achieve a significant speedup and significantly smaller shield sizes when introducing a fail-safe mode. We also investigated how the synthesis time increased with an increasingly larger bound *b*. For Property 8 and Property 6, the run time and shield size remained small even for large automata. For Property 10, the run time and shield size grew faster. The reason lies in the fact that for some properties, an error by the design results in a large set of states to be monitored, while for other properties, this set stays rather small.

## Shield synthesis for human–autonomy interaction

**Fig. 13:**
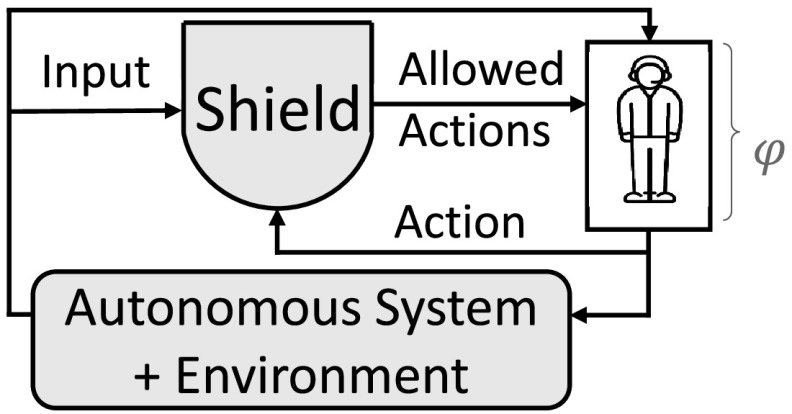
Preemptive shielding

In the second part of the paper, we consider shielding a human operator who works with an autonomous system. In the context of shielding a human operator we often refer to outputs of the human operator as actions selected by the operator. Shielding a human operator instead of shielding a reactive system requires two innovations in the shielding procedure. (1) In the case of shielding reactive systems, the shield is attached *after* the system and corrects the output of the system—see Fig. 1. In contrast, when shielding human operators we attach the shield *before* the operator and the shield restricts the possible actions of the operator—see Fig. 13. We call these types of shields *preemptive shields*. (2) When shielding a human operator, it is necessary to provide simple and intuitive explanations to the operator for the interferences of the shield. We call shields that are able to provide such explanations *explanatory shields*. In the next sections, we address these two innovations in more detail.

### Preemptive shields

Consider a setting in which a human operator controls an autonomous reactive system: in every time step, the environment and the system provide an input (sensor measurements, state information) to the operator, then the operator selects the next action for the system to be executed, the system executes the selected action, and the system and the environment move to the next state.

The human operator has to select actions in such a way that a given safety specification $$\varphi = (Q, q_{0}, \varSigma _I\times \varSigma _O, \delta , F)$$ with $$\mathcal {A}=\varSigma _O$$ is the set of available actions, is met. In any given situation an action $$a \in \mathcal {A}$$ is called $$unsafe $$ if after which the system and the environment can force the specification to be violated. Otherwise, an action is called *safe*. We modify the loop between the human operator and the system, depicted in Fig. 13. The shield is implemented *before* the human operator and acts each time the operator is to make a decision by providing a list of safe actions. The task of the shield is to modify the set of available actions of the human operator in every time step such that only safe actions remain.

The interaction between the environment, the autonomous system, the human operator, and the shield is as follows: At every time step, the shield computes a set of all safe actions $\{a_1, \dots , a^k \}$, i.e., it takes the set of all actions available, and removes all unsafe actions that would violate $\varphi$ (in the worst case over future inputs). The human operator receives this list from the shield, and picks an action $a\in \{a_1, \dots , a^k \}$ from it. The autonomous system executes action *a*, the system and the environment move to the next state and provide the next input to the shield and the operator.

More formally, for a preemptive shield $\mathcal {S}= (Q^S, q_0^S, \varSigma _I^S, \varSigma _O^S, \delta ^S, \lambda ^S)$, we have $\varSigma _O^S=2^\mathcal {A}$, as the shield outputs the set of actions for the human operator to choose from for the respective next step. The shield observes the same inputs as the human operator. For selecting the next transition of the finite-state machine that represents the shield, it also makes use of the action actually chosen by the human operator. So for the input alphabet of the shield, we have $\varSigma _I^S=\varSigma _I\times \mathcal {A}$.

When shielding a human operator, preemptive shielding provides many advantages. First, the shield ensures that a given safety specification is assured. Second, the shield is least restrictive to the operator, since it allows the operator to pick any action, as long as it is safe.

#### Synthesizing preemptive shields

Given is a setting in which a human operator has to control an autonomous system in an unknown environment while satisfying a safety specification $\varphi = (Q, q_{0}, \varSigma _I\times \varSigma _O, \delta , F)$ with $\mathcal {A}=\varSigma _O$ is the set of available actions the human operator can choose from. Starting from $\varphi$, our shield synthesis procedure is as follows:

1. Consider $\varphi$ as a safety game and compute its winning region $W\subseteq F$ so that every system $\mathcal {D}\models \varphi$ must produce outputs such that the next state of $\varphi$ stays in *W*.
2. We translate *Q* and *W* to a reactive system $\mathcal {S}= (Q^S, q_0^S, \varSigma _I^S, \varSigma _O^S, \delta ^S, \lambda ^S)$ that constitutes the shield with the state space $Q^S=Q$, the initial state $q_0^S=q_0$, the input $\varSigma _I^S=\varSigma _I\times \mathcal {A}$, the output $\varSigma _O^S=2^\mathcal {A}$, the transition relation $\delta ^S$ and the output function $\lambda ^S$ such that
  $$\begin{aligned} \delta ^S(q, {\sigma _I}, a)= & {} \delta (q, {\sigma _I}, a) \text { for all } q \in Q, {\sigma _I}\in \varSigma _I, a \in \mathcal {A}\text { and} \\ \lambda ^S(q, {\sigma _I})= & {} \{a \in \mathcal {A} \mid \delta (q, {\sigma _I}, a) \in W \} \text { for all } q \in Q, {\sigma _I}\in \varSigma _I. \end{aligned}$$

**Fig. 14:**
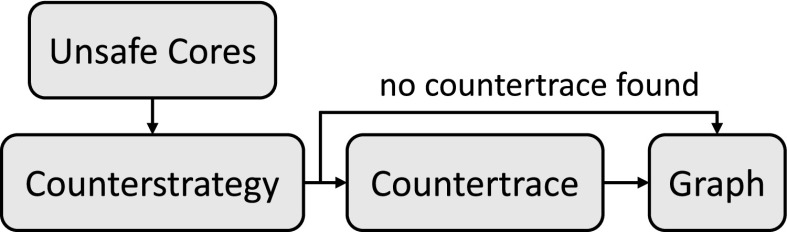
Procedure to explain unsafe outputs

The shield allows all actions that are guaranteed to lead to a state in *W*, no matter what the next input is. Since these states, by the definition of the set of winning states, are exactly the ones from which the system player can enforce not to ever visit a state not in *F*, the shield is minimally interfering. It disables all actions that may lead to an unsafe state.

### Explanatory shields

Explanatory shields provide a simple diagnosis to the operator and explain why certain actions are unsafe in the current situation. They are particulary useful in cases in which $\varphi$ is very complex, e.g., consists of thousands of states, and it is difficult for the operator to comprehend why the shield had to interfere. To explain unsafe actions, we propose to use techniques for debugging formal specifications [23].

Figure 14 depicts the flow of our method to explain unsafe actions. First, we compute a minimal set of properties $\varphi _i$ and signals that on their own are sufficient to show that an action is unsafe in a given situation. We call this part of the specification that contains the root causes for an action to be unsafe an *unsafe core*. Using an unsafe core, we compute a counterstrategy to explain why the action is indeed unsafe. Since counterstrategies may be large, we apply a heuristic to search for a countertrace, i.e., a single input trace which necessarily leads to a specification violation. Finally, we provide the operator with a graph that summarizes all plays that are possible against the counterstrategy (or the countertrace, in case the heuristic succeeds). Next, we will detail these steps.

#### Unsafe cores

Understanding why an action for a given state-input pair is unsafe may be difficult, but often only a small part of the specification is responsible. Removing extraneous parts from the specification gives a specification that still forbids the action but is much smaller and thus easier to understand. We call this part of the specification the *unsafe core* for a given action in a specific situation, i.e., a state-input combination.

Typically, a safety specification $\varphi$ is composed of several safety properties $\varphi =\{\varphi _1\dots \varphi _l\}$, where each $\varphi _i$ defines a relatively self contained and independent aspect of the system behavior. Our goal is to identify minimal sets of properties $\varphi _i$ that explain the unsafe action on their own.

##### Definition 10

*(Unsafe Core)* Let $\varphi =\{\varphi _1,\dots ,\varphi _l\}$ with $\varphi _1 \times \dots \times \varphi _l= (Q, q_{0}, \varSigma _I\times \varSigma _O, \delta , F)$ be a safety specification. A subset $\phi \subseteq \varphi$ defines an *unsafe core* in state $q \in Q$ and for an input ${\sigma _I}\in \varSigma _I$ and an output ${\sigma _O}\in \varSigma _O$ if executing ${\sigma _I}{\sigma _O}$ from *q* results in a next state $q' \in Q$ outside the winning region of $\varphi$ (when $\varphi$ is interpreted as a game), and there is no strict subset of $\varphi$ for which the same holds.

In the computation of unsafe cores, one can also remove signals from the specification in addition to properties. Removal of signals allows the operator to focus on those signals that are relevant for the problem at hand. It also simplifies the following counterstrategies, making them free of irrelevant signals.

Unsafe cores are very similar to unrealizable cores [12], i.e., parts of $\varphi$ that are unrealizable on their own. Computing unsafe cores reduces to computing unrealizable cores, which can efficiently be computed by computing minimal hitting sets [26].

#### Counterstrategies and countertraces

Suppose we are given a safety specification $\varphi = (Q, q_{0}, \varSigma _I\times \varSigma _O, \delta , F)$ to be enforced by $\mathcal {S}$. Explaining why an output ${\sigma _O}$ from a state *q* and an input ${\sigma _I}$ is unsafe boils down to presenting that from $q'=\delta (q,{\sigma _I}, {\sigma _O})$, there exists a strategy for selecting future inputs such that a state outside *F* is reached eventually, no matter how future outputs are selected. This strategy for selecting such inputs is called a counterstrategy. Understanding the counterstrategy implies understanding why the output is unsafe.

In general, a counterstrategy cannot be presented as a single trace of inputs, since inputs may depend on previous outputs. The dependencies can become quite complex, especially for large specifications, which makes it difficult for the operator to comprehend which environment behavior leads to unsafety. The operator may prefer one single trace of inputs such that there are no future outputs able to satisfy $\varphi$. Such a trace is called a countertrace. Unfortunately, a countertrace does not always exist. Even if one exists, its computation is often expensive. We therefore propose to use a heuristic to compute countertraces as presented in [23].

### Case study on UAV mission planning

In this section, we apply shields on a scenario in which an unmanned aerial vehicle (UAV), controlled by a human operator, must maintain certain properties while performing a surveillance mission in a dynamic environment. We show how a preemptive explanatory shield can be used to enforce the desired properties and to provide feedback to the operator for any restrictions on the commands available.

To begin, note that a common UAV control architecture consists of a ground control station that communicates with an autopilot onboard the UAV [11]. The ground control station receives and displays updates from the autopilot on the UAV’s state, including position, heading, airspeed, battery level, and sensor imagery. It can also send commands to the UAV’s autopilot, such as waypoints to fly to. A human operator can then use the ground control station to plan waypoint-based routes for the UAV, possibly making modifications during mission execution to respond to events observed through the UAV’s sensors. However, mission planning and execution can be workload intensive, especially when operators are expected to control multiple UAVs simultaneously [14]. Errors can easily occur in this type of human–automation paradigm, because a human operator might neglect some of the required safety properties due to high workload, fatigue, or an incomplete understanding of exactly how a command is executed.

As the mission unfolds, waypoint commands will be sent to the autopilot. A shield that monitors the inputs and restricts the set of available waypoints would be able to ensure the satisfaction of the desired properties. Additionally, a shield should explain any restrictions it makes in a simple and intuitive way to the operator.

**Fig. 15:**
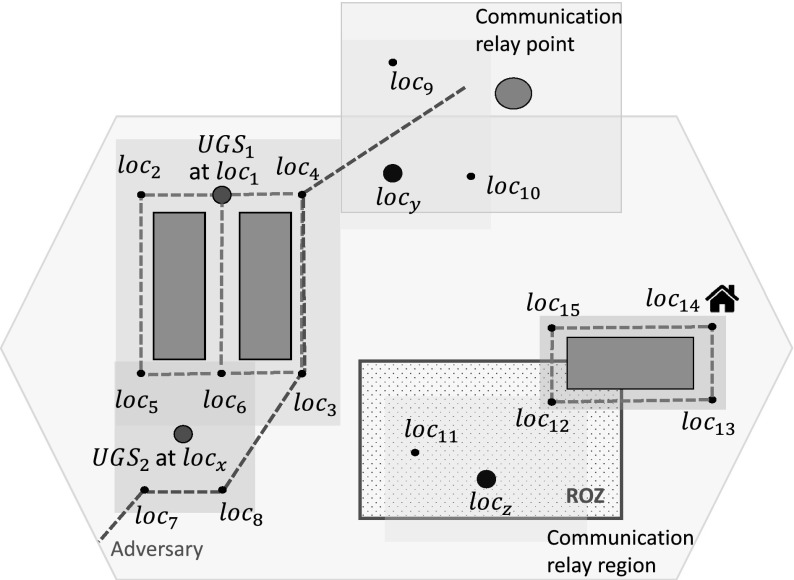
A map for UAV mission planning

Consider the mission map in Fig. 15 [21], which contains three tall buildings (illustrated as blue blocks), over which a UAV should not attempt to fly. It also includes two unattended ground sensors (UGS) that provide data on possible nearby targets, one at location $loc_1$ and one at $loc_x$, as well as two locations of interest, $loc_y$ and $loc_z$. The UAV can monitor $loc_x$, $loc_y$, and $loc_z$ from several nearby vantage points. The map also contains a restricted operating zone (ROZ), illustrated with a red box, in which flight might be dangerous, and the path of a possible adversary that should be avoided (the pink dashed line). Inside the communication relay region (large green area), communication links are highly reliable. Outside this region, communication relies on relay points with lower reliability.

Given this scenario, properties of interest include:

1. *Adjacent waypoints* The UAV is only allowed to fly to directly connected waypoints.
2. *No communication* The UAV is not allowed to stay in a zone with reduced communication reliability and has to leave this zone within 1 time step.
3. *Restricted operating zones* The UAV has to leave a ROZ within 2 time steps.
4. *Detected by an adversary* Locations on the adversary’s path cannot be visited more than once over any window of 3 time steps.
5. *UGS* (a) If $UGS_1$ reports a target, the UAV should visit $loc_1$ within 7 steps. (b) If $UGS_2$ reports a target, the UAV should visit $loc_5$, $loc_6$, $loc_7$, or $loc_8$ within 7 steps.
6. *Refuel* Once the UAV’s battery is low, it should return to a designated landing site at $loc_{14}$ within 10 time steps.

The task of the shield is to ensure these properties during operation. In this setting, the human operator responds to mission-relevant inputs, e.g., in this case data from the UGSs and a signal indicating whether the battery is low. In each step, the operator sends the next waypoint to the autopilot, which is encoded in a bit representation via outputs $l_4$, $l_3$, $l_2$, and $l_1$. The shield is implemented *before* the operator. It monitors mission inputs, and provides a list of safe waypoints to the operator each time the operator is going to select a next one. This list restricts the choices of the operator.

**Fig. 16:**
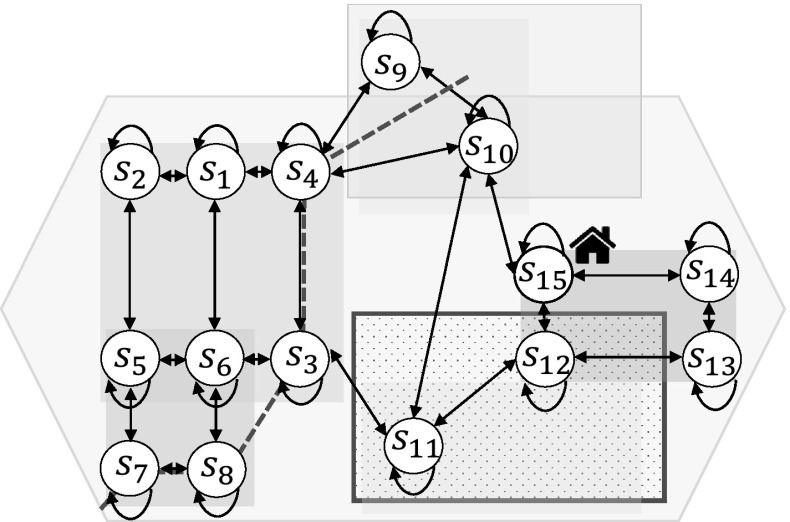
Safety automaton of Property P1

We represent each of the properties by a safety automaton, the product of which serves as the shield specification $\varphi$. Figure 16 models the “connected waypoints” property, where each state represents a waypoint with the same number. Edges are labeled by the values of the variables $l_4\dots l_1$. For example, the edge leading from state $s_5$ to state $s_6$ is labeled by $\lnot l_4 l_3 l_2 \lnot l_1$. For clarity, we drop the labels of edges in Fig. 16. The automaton also includes an error state, which is not shown. Missing edges lead to this error state, denoting forbidden situations.

For our experiments, we used the six Properties P1-P6 as safety specification $\varphi _1, \dots , \varphi _6$. We synthesized preemptive shields as described in Sect. 4.1.1. To provide explanations for unsafe actions, we computed the unsafe core of the properties $\{\varphi _1, \dots , \varphi _6\}$. All results are summarized in Table 7. The left four columns list the set of properties and the number of states, inputs, and outputs of their product automata, respectively. The last column lists the time to synthesize the explanatory preemptive shield. Note that in order to compute the unsafe cores, it is necessary to compute shields for all possible subsets of properties. The synthesis time given in Table 7 is the total time including the creation of all shields used to compute the unsafe cores.

All computation times are for a computer with an Intel Xeon 4.0GHz CPU and 16GB RAM running a 64-bit distribution of Linux.

**Table 7:** Results for UAV experiments

Property	|*Q*|	|*I*|	|*O*|	Time (s)
1	16	0	4	0.01
1 $+$ 2	16	0	4	0.13
1 $+$ 2 $+$ 3	19	0	4	0.27
1 $+$ 2 $+$ 3 $+$ 4	23	0	4	0.92
1 $+$ 5a	84	1	4	0.9
1 $+$ 5a $+$ 2	84	1	4	3.95
1 $+$ 5a $+$ 2 $+$ 3	100	1	4	12.48
1 $+$ 5b	64	1	4	0.5
1 $+$ 5b $+$ 2	64	1	4	1.97
1 $+$ 6	115	1	4	1.6

**Fig. 17:**
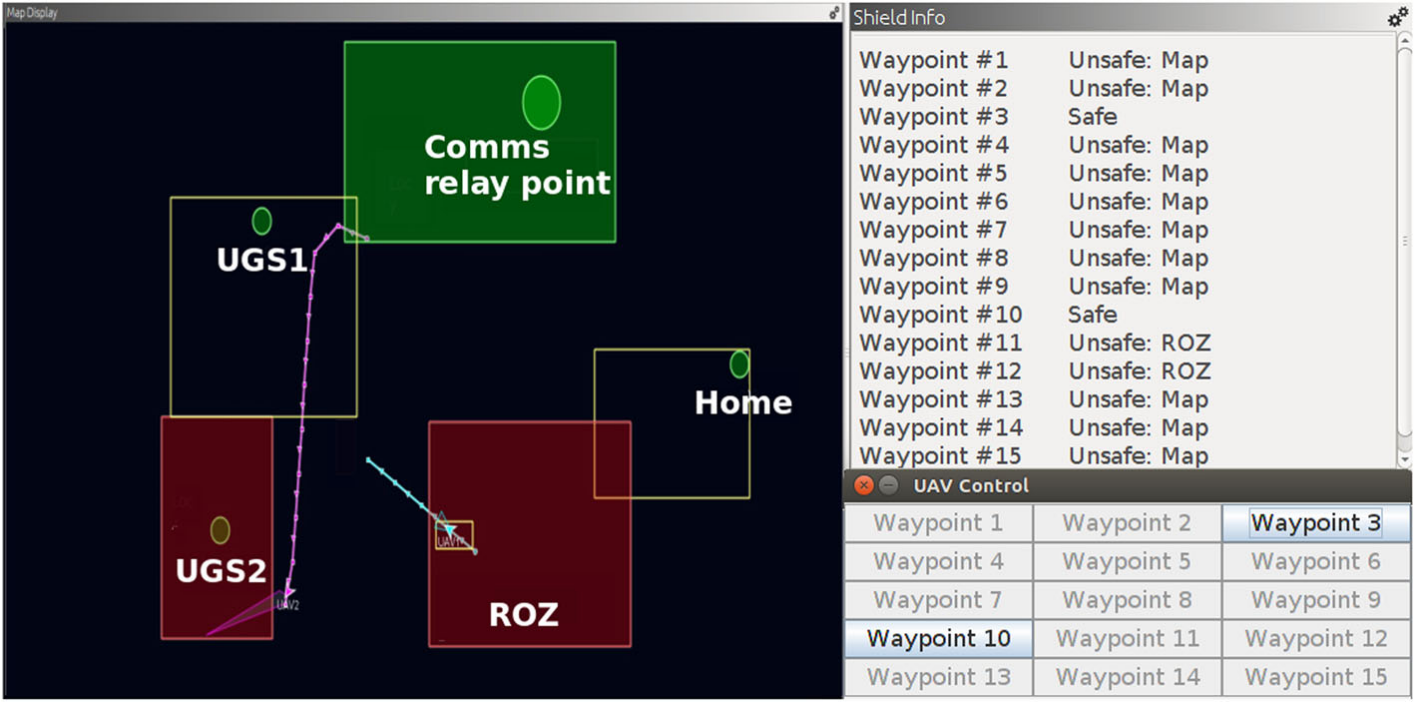
Simulation of explanatory preemptive shield developed on AMASE

We integrated our shields in the AMASE multi-UAV simulator [15]. AMASE is a flight simulation environment, which models UAVs using a kinematic flight dynamics model that includes environmental effects (e.g., wind) on performance. Fig. 17 visualizes the map of Fig. 15 using AMASE and shows one UAV (in blue), currently in the ROZ at location $loc_{11}$ and controlled by the human operator, and a second adversarial UAV (in pink) on its way to $loc_8$. We integrate a shield to ensure the six safety properties P1–P6. On the right-hand side of the graphical interface, the operator can select the next waypoint for the controlled UAV. In the current situation, adjacent waypoints for the controlled UAV are $loc_3$, $loc_{10}$, $loc_{11}$, and $loc_{12}$, with $loc_3$ and $loc_{10}$ being outside the ROZ. The shield disables all other waypoints because the UAV is only allowed to fly to directly connected waypoints, according to Property P1. Assume that the controlled UAV already spent two time steps in the ROZ. Therefore, it has to leave the ROZ in the next time step, according to Property P3, and the shield disables the waypoints at location $loc_{11}$ and $loc_{12}$ as well. The explanation for any restrictions made by the shield are illustrated in the upper right corner of the GUI.

## Related work

Our work builds on synthesis of reactive systems [7, 33] and reactive mission plans [17] from formal specifications, and our method is related to synthesis of robust [4] and error-resilient [18] systems. However, our approach differs in that we do not synthesize an entire system, but rather a shield that considers only a small set of properties and corrects the output of the system at runtime. Li et al. [25] focused on the problem of synthesizing a semi-autonomous controller that expects occasional human intervention for correct operation. A human-in-the-loop controller monitors past and current information about the system and its environment. The controller invokes the human operator only when it is necessary, but as soon as a specification will be violated, such that the human operator has sufficient time to respond. Similarly, our shields monitor the behavior of systems at run time and interfere as little as possible. Our work relates to more general work on runtime enforcement of properties [20], but shield synthesis is the first appropriative work for reactive systems, since shields act on erroneous system outputs immediately without delay.

This paper extends our previous work on shield synthesis [8, 22]. In [8] we defined the general framework for solving the shield synthesis problem for reactive systems, and proposed the *k*-stabilizing shield synthesis procedure that guarantees recovery in *k* steps. Admissible shields were proposed in [22]. Here we assume that systems generally have cooperative behavior with respect to the shield, i.e., the shield ensures a finite number of deviations if the system chooses certain outputs. This is similar in concept to cooperative synthesis as considered in [6], in which a synthesized system has to satisfy a set of properties (called guarantees) only if certain environment assumptions hold. The authors present a synthesis procedure that maximizes the cooperation between system and environment for satisfying both guarantees and assumptions as much as possible. This work extends the previous work on shield synthesis by (1) discussing how liveness properties of the design can be preserved when shielded, (2) introducing a procedure for shielding the decisions of a human operator, and (3) presenting new experiments.

## Conclusion

In this paper, we have presented automated synthesis of shields for reactive systems. Given a set of safety specifications, a shield monitors the inputs and outputs of the reactive system and corrects erroneous output values at runtime. The shield deviates from the given outputs as infrequently as it can and recovers to hand back control to the system as soon as possible. We provided theoretical and algorithmic background for three concrete instantiations of the shield concept: (1) *k*-stabilizing shields that guarantee recovery in a finite time. (2) Admissible shields that attempt to work with the system to recover as soon as possible. (3) An extension of *k*-stabilizing and admissible shields in which erroneous output values are corrected such that liveness properties of the system are preserved. The results from numerical experiments illustrate the scalability and effectiveness of the shield synthesis methods. In addition to these numerical experiments on existing benchmarks, we illustrated the broad applicability of shielding in a case study on human–autonomy interactions. We considered shielding the decisions of a human operator who works with an autonomous system. In this setting, we pursued shields that not only enforce correctness but also support the operator with intuitive explanations of the sources of potential wrong behavior and any restrictions caused by the shield. We presented results involving mission planning for unmanned aerial vehicles. We foresee a number of potential future research directions. These include shielding adaptive systems whose functionality evolves over time and systems that implement various learning algorithms. In general, we expect the conceptual simplicity of shielding to be offer a novel approach to develop artificially intelligent systems with provable safety and correctness guarantees.

## Footnotes

1
http://www.iaik.tugraz.at/content/research/design_verification/others/ .
